# An integrated framework to identify and characterize regional‐scale insect dispersal

**DOI:** 10.1002/eap.70230

**Published:** 2026-04-20

**Authors:** Felipe Dargent, Megan S. Reich, Marrissa Miller, Kala Studens, Nilofar Benvidi, Kerry Perrault, Joshua Aibueku, Brent Holmes, Clement P. Bataille, Jean‐Noël Candau

**Affiliations:** ^1^ Natural Resources Canada – Canadian Forest Service, Great Lakes Forestry Centre Sault Ste. Marie Ontario Canada; ^2^ Department of Earth and Environmental Sciences, Advanced Research Complex Building University of Ottawa Ottawa Ontario Canada; ^3^ Department of Biology University of Ottawa Ottawa Ontario Canada; ^4^ Department of Biology Carleton University Ottawa Ontario Canada; ^5^ Purdue University, Department of Forestry and Natural Resources Purdue University West Lafayette Indiana USA

**Keywords:** δ^34^S, *Choristoneura fumiferana*, HYSPLIT trajectory model, insect dispersal, isoscape, isotope‐based geographic assignment, pest management, spruce budworm moth, sulfur isotopes

## Abstract

Forest pest insects cause major socio‐economic impacts, global losses of millions of dollars, and ecosystem changes. A key challenge for their management is tracing regional dispersal events critical to outbreak dynamics. We developed an integrated tracing framework for pest insects by combining isotope geolocation, ecological data, and atmospheric modeling, and applied this framework to the eastern spruce budworm moth (*Choristoneura fumiferana*), the most severe defoliator of the North American boreal forest, to trace outbreak dispersal events. We first generated a North American model of bioavailable sulfur isotope (δ^34^S) variation in space (isoscape) and then calibrated it to spruce budworm tissues of known origin. We then used an automated trap network with high temporal resolution to collect samples and identify potential immigration events of eastern spruce budworm to Nova Scotia, Canada. Finally, we traced the natal origin of these immigrants by sequentially integrating high‐probability regions of origin derived from δ^34^S values and estimated migration routes derived from biologically constrained atmospheric transport models. We find that this integrated framework allows us to narrow down the region of pest origins, restricting it to a few possible locations and demonstrating long‐distance dispersal of spruce budworm across ~400 km over the Gulf of St. Lawrence, Quebec. Our framework demonstrates that combining isotopic data with ecological indicators and atmospheric transport modeling offers improved resolution and understanding of insect dispersal ecology. This approach is transferable to trace other migratory insect species to address conservation, agriculture, and bio‐surveillance needs in the context of global environmental change.

## INTRODUCTION

Insect pests are a major economic and societal challenge (Bradshaw et al., 2016). Each year they cost the global economy hundreds of billions of dollars in crop losses (Culliney, 2014; FAO, 2021). These costs are expected to increase in the future as human populations and demand for agricultural and forestry products continue to grow, climate change exacerbates the frequency and extent of forest insect outbreaks (Jactel et al., 2019) and crop losses (Deutsch et al., 2018), and changing wind patterns facilitate pest dispersal to new regions (Harvey et al., 2023). The scale and magnitude of these impacts is closely tied to each species' dispersal capacity because it influences the rate of pest spread, their recurrence, and their likelihood of establishment (Asplen, 2018; Heimpel & Asplen, 2011).

Successful management strategies to forecast and mitigate pest risks to ecosystems, agriculture, and human societies require, first, developing effective tools to evaluate dispersal (Coulson et al., 1993). Such tools should provide accurate spatiotemporal knowledge of the source, trajectory, and endpoint of pest movements and allow the identification of causal mechanisms that trigger insect movement. Yet, several factors have limited our ability to identify and characterize insect dispersal events. First, the vast magnitude and frequency of insect dispersal have only been demonstrated recently (Satterfield et al., 2020), and they incorporate a broad diversity of movement strategies (Chapman et al., 2015; Satterfield et al., 2020). Second, typical animal tracking methods (e.g., radio telemetry) are challenging to implement on insects due to their low body mass. Third, population level tracking using mark and recapture can be effective for species with recurrent routes and long lifespans (e.g., monarch butterflies—Mouritsen et al., 2013) or when tracking short‐distance movement (e.g., spruce budworm moths <100‐m dispersal—Sanders, 1983, Kipp & Lonergan, 1992), but becomes exponentially challenging at large spatial scales due to the short lifespans, enormous populations, and often stochastic dispersal routes (El Sheikha, 2019). Fourth, while recent tracing innovations, such as radar and population genetics, show promise, their successful implementation can be highly context‐specific. For example, radar technology requires extensive infrastructure and can only detect massive migration events (e.g., Shamoun‐Baranes et al., 2014), whereas migration and outbreaks often increase gene flow erasing the genetic population structure (e.g., Lumley et al., 2020). An integrated approach combining isotopic tracers that are naturally incorporated into insect tissues, coupled with atmospheric dispersal models and automated traps, could potentially circumvent many of these limitations.

Isotopes have been used for decades to reconstruct long‐distance insect migration because they function as intrinsic markers of geographic origin (Quinby et al., 2020). Some isotopes vary predictably on the landscape, and when insect larvae develop at a given location, they inherit a local isotope “fingerprint” on tissues metabolized at that site (Quinby et al., 2020). If those tissues have limited metabolic recycling (e.g., wings) (Lindroos et al., 2023) and if the studied isotope varies predictably in space (Reich et al., 2023), the tissue can preserve the isotopic “fingerprint” of its site of natal origin. By comparing the isotopes of a migratory individual to a reference map predicting isotopes on the landscape (i.e., an isoscape), one can then estimate the origin of that individual. Hydrogen and strontium isotopes have been successfully used as geolocators for insects (e.g., Reich et al., 2021), but isotopes of other elements could increase insect geolocation resolution. Sulfur isotopes (referred to as δ^34^S) are promising because: (1) they are present in measurable amounts in many protein‐rich insect tissues (Tcherkez & Tea, 2013), and (2) they display predictable spatial gradients on the landscape (Appendix S1: Figure S1), with δ^34^S values decreasing from the coast towards inland regions due to the decreasing deposition of ^34^S‐enriched marine sulfates (e.g., in leaves—Sparks et al., 2019; Tarrant & Richards, 2024; human hair—Bataille, Chartrand, et al., 2020; Valenzuela et al., 2011; and moths—Newton, 2021).

Atmospheric particle dispersal models are increasingly popular to trace insect dispersal because they provide fine resolution estimates of trajectories and are cost‐effective (NOAA, 2020) relative to traditional methods such as mark–recapture and population genetic tools. These models use time‐dependent gridded meteorological data and atmospheric physics to calculate the transport and diffusion processes of air parcels in the atmosphere and can be applied to simulate the release, transport, and deposition of particles, including wind‐transported insects (e.g., Stein et al., 2015). Their precision can be enhanced by incorporating insect behavioral and ecological parameters such as flight phenology, biophysical constraints, mass‐dependent effects, and population dynamics (e.g., Otuka et al., 2023). However, a prerequisite to apply these models effectively is to know the location and time of the insect's arrival (Preti et al., 2021). Integrating automated trap networks and isotope geolocation could fill this gap because: (1) automated traps equipped with cameras could provide this missing temporal context (e.g., time of capture), and (2) isotopes could provide, independently, the missing information on the location of natal origin.

The capability of tracing insect pests at high resolution is currently limited, and its absence constrains the development of more effective management and decision‐support tools. The integrated approach we propose would enable earlier and more accurate detection of dispersal events, help predict the direction and rate of outbreak fronts, and support a shift from reactive responses to proactive and predictive pest management. Moreover, identifying the most probable source regions or dispersal “hotspots” would provide key ecological insights into the drivers of movement, ultimately improving both forecasting and long‐term surveillance strategies.

In this study, we use the eastern spruce budworm moth (*Choristoneura fumiferana* Clemens, hereafter spruce budworm) (Lepidoptera: Tortricidae) as a model species to test this integrative‐tracing approach. This species is excellent for conducting these tests because dispersal is likely a critical mechanism of outbreak spread of this pervasive and severe defoliator (MacLean, 2019; Morris, 1963). During outbreaks, population density increases by several orders of magnitude, causing extensive defoliation of spruce (*Picea* sp.) and balsam fir (*Abies balsamea*). Resource scarcity and high population densities then initiate mass emigration from outbreak centers (Morris, 1963; Régnière & Nealis, 2007) into low‐density populations hundreds of kilometers away (Barnéoud et al., 2025; Greenbank et al., 1980) and drive outbreak expansion (Morris, 1963).

Effective management of these outbreaks requires early warning systems capable of forecasting immigration into vulnerable areas to enable targeted preventive measures that anticipate and stop outbreak spread (Johns et al., 2019). It also requires increased knowledge of the biotic and abiotic drivers of take‐off (emigration) and landing (immigration) that contribute to outbreaks, to develop mechanistic models of outbreak spread. Neither of those directions can be advanced without tools to trace dispersal. As a result, current approaches rely on expensive and time‐consuming in situ monitoring of vast forest areas and reactive management approaches (Natural Resources Canada, 2021).

To test this integrated approach for insect geolocation, we first developed a foliar δ^34^S (δ^34^S_foliar_) isoscape for North America and calibrated it into a δ^34^S spruce budworm (δ^34^S_moth_) isoscape with wild‐reared spruce budworm moths. We then leveraged automated pheromone traps across eastern Canada to collect immigrant moths from previously identified immigration events (Dargent et al., 2023) and used immigrant δ^34^S values and δ^34^S_moth_ isoscapes to model isotope‐based probabilistic assignments of natal origins. Finally, we simulated potential dispersal pathways from defoliated regions, selected those that reached the capture locations of these immigrants, and overlaid the results with the δ^34^S natal origins to refine our origin predictions. We use these findings to discuss the broader impacts of this integrated framework as a tool to inform insect pest management.

## METHODS

### Foliar sulfur isoscape

#### Foliar sample collection

**FIGURE 1:**
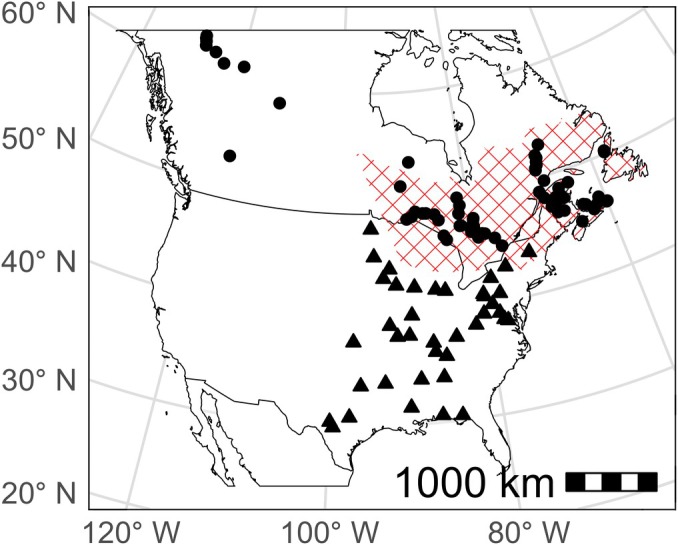
Map depicting the sampling locations of spruce and balsam fir (points) and milkweed (triangles). The red crosshatched region delimits the range of eastern spruce budworm (Rauchfuss & Ziegler, 2011).

Since plants are the primary source of sulfur for folivore insect larvae, we expect a predictable relationship between local δ^34^S_foliar_ and the δ^34^S of adult insects (δ^34^S_moth_) that fed on those plants as larvae. Thus, a δ^34^S_moth_ isoscape could be readily constructed from a δ^34^S_foliar_ isoscape (Hobson, 2019). To develop the δ^34^S_foliar_ isoscape for North America, we collected mature needles from the host plants of spruce budworm, balsam fir (*A. balsamea*) and spruce (*Picea* sp.), across mostly eastern Canada, from sites encompassing a broad range of environmental, geological, geographic, anthropogenic, and climatic conditions. These samples were collected between 2019 and 2023 by collaborators and volunteers at 157 sites, and their latitude and longitude were recorded. At 77 of these sites, we collected branches at 1.5 m above the ground from three trees less than 10 m apart. At the remaining 80 sites, only one tree was sampled. In the laboratory, each branch was subsampled and, when possible, species was recorded before the samples were stored in kraft envelopes in a dry room. To cover a broader geographic range, and because species‐specific fractionation effects on sulfur isotope composition are expected to be small at a continental scale relative to environmental and geographic drivers, we also analyzed δ^34^S_foliar_ in common milkweed (*Asclepias syriaca*) collected from 39 sites across the United States in the summer of 2018 (see Reich et al., 2021). In total, samples were collected from 197 sites (Pinaceae *n* = 157, milkweed *n* = 39) between 2018 and 2023 (most milkweed samples were collected in 2018; Figure 1).

We tested the possible intra‐site δ^*3*4^S_foliar_ variation caused by tree species differences by collecting and comparing spruce and balsam fir needles from the same sites (*n* = 8). Additionally, since spruce budworm prefers feeding on more nutritious immature (i.e., young) foliage (Fuentealba & Bauce, 2012), we collected both young needles and fully mature needles from the same trees at seven sites and compared their isotopic composition.

#### Foliar sample preparation and isotope analysis

Needles were aggregated by site, rinsed twice with deionized water to remove surficial particles, and dried at 50°C for 48 h. Milkweed leaves were aggregated by site and rinsed with deionized water in an ultrasonic bath and then dried at 60°C for 7 days. Dry needles and leaves were ground to a fine powder and stored in glassine envelopes. The differences in milkweed preparation, relative to spruce and balsam fir, reflect their use in different projects; these conditions are not expected to influence sulfur isotope composition.

Sulfur isotopes were analyzed at the Jan Veizer Stable Isotope Laboratory at the University of Ottawa. We micro‐weighed a target mass of 60 ± 6 mg of conifer needle powder (S% = 0.09 ± 0.03) and 25 ± 2.5 mg of milkweed leaf powder (S% = 0.51 ± 0.18) into tin capsules. Samples were combusted through an Elemental Analyzer Isotope Cube in S mode (Elementar, Germany), with combusted SO_2_ from the sample carried to a Delta Plus XP IRMS (ThermoFinnigan, Germany) equipped with a ConFlo IV. The ^34^S/^32^S ratios were obtained from *m/z* 48, 49, and 50 of SO^+^ produced from SO_2_. Standards used for three‐point calibration were sulfide‐based IAEA‐S1 (argentite, δ^34^S = −0.3‰ ± 0.03‰), IAEA‐S2 (argentite, δ^34^S = 22.7‰ ± 0.08‰), and IAEA‐S3 (argentite, δ^34^S = −32.6‰ ± 0.08‰) international standards (International Atomic Energy Agency, 2020; IAEA, Vienna, Austria); AG‐2 (argentite, δ^34^S = −0.6‰) was used as an internal blind standard. Sulfur isotope values are reported to the international scale Vienna Canyon Diablo Troilite (VCDT). The measured values for AG‐2 among runs varied between −0.4‰ and −1.1‰ (among run average = −0.8‰ ± 0.3‰, *n* = 9), and were within the laboratory long‐term reported value of −0.6‰ and uncertainty of 0.3‰. Analytical precision of these measurements is based on the reproducibility of 22 foliar powder samples, which had a mean difference between duplicates of 0.16‰ ± 0.15‰.

#### Foliar isoscape modeling

We first built a δ^34^S_foliar_ isoscape using random forest regression following the methods of Bataille, Crowley, et al. (2020), Bataille et al. (2021), and Reich et al. (2024). The model incorporated the newly produced δ^34^S data from foliar samples across eastern North America and geospatial variables representing geological, atmospheric, climatic, or environmental controls of sulfur isotope cycling. The assembled covariates have been previously described (Reich et al., 2024; Appendix S1: Table S1). The tuning and optimization of the random forest model has been described in previous work (Bataille, Crowley, et al., 2020). Briefly, we used the “tuneRF” function of the *caret* package (Kuhn, 2008) using root mean square error (RMSE) through a 10‐fold cross‐validation approach with five repetitions, where 80% of the data was used for training at each iteration. We used the *VSURF* package (Genuer et al., 2015) to maximize model performance while minimizing the number of predictors. Partial dependence plots were also used to evaluate the relationship between δ^34^S_foliar_ values and the selected predictors. We estimated model uncertainty using quantile random forest to calculate a 68.27% prediction interval (Funck et al., 2021), which we used to then estimate the SD (σ) of predictions at each pixel with: 𝜎 ≈ (0.841) − (0.159). Isoscapes were then trimmed by “masking” areas for which predictor values were beyond the training set range to avoid extrapolation. To assess the sensitivity of the isoscape to plant taxon, models were constructed for the conifer and milkweed datasets separately (Appendix S1: Figure S2). However, the performance, absolute values, and spatial patterns were comparable between subsets, so we only report the combined model in the main manuscript.

### Spruce budworm moth‐calibrated δ ^34^S isoscape

#### Known‐origin moth sample collection

The relationship between δ^34^S_foliar_ and δ^34^S_moth_ values for spruce budworm is unknown, although previous studies have shown limited overall fractionation between resource and tissue in invertebrates (mean Δ^34^S = +0.6‰ ± 0.3‰, nonsignificantly different from 0) and a slight depletion of ^34^S relative to the food source in other animals (Raoult et al., 2024). As spruce budworm ingest plants and liquid only during the larval stages and have a short adult lifespan of ~10 days (Miller, 1963), the isotopic composition of adult tissues likely reflects that of the larval diet.

To assess the relationship between δ^34^S_foliar_ and δ^34^S_moth_ values and account for potential trophic level isotopic fractionation, we collected spruce budworm from up to three adjacent trees at 19 sites in Ontario, six sites in Newfoundland, and three sites in New Brunswick (“known‐origin” samples—Appendix S1: Table S2). Following Contina et al. (2022), the sites were selected to include extreme values and a broad range of environmental conditions known to influence sulfur isotope cycling (e.g., Bataille et al., 2021; Brlík et al., 2023). We favored collecting field samples over rearing spruce budworm on isotopically distinct diets to avoid potential bias in how sulfur in the diet is incorporated into the tissues and to better account for the potential incorporation of atmospheric deposition on ingested needles. For each known‐origin site (except those in New Brunswick, for which we had no foliar samples), we collected foliar samples from the same trees from which we sampled spruce budworm, or within 10 m of budworm collection (NL), and pooled them for foliar analysis as described in *Foliar sample collection* section. To ensure they were true locals, most known‐origin moths in our dataset were collected in the field as pupae and allowed to eclose in captivity in paper bags at roughly 22°C (laboratory temperature). We recorded the geographic coordinates, collection date, site description, and match between plant and moth samples. To assess intra‐site δ^34^S_moth_ variations, we aimed to obtain three to five adults per site, but this was not possible at all sites, due to low density of budworm, failure to eclose, and attacks by Hymenoptera parasitoids (Appendix S1: Table S2).

#### Moth sample preparation and isotope analysis

Each moth was cleaned in a 2:1 v/v chloroform:methanol solution in three successive washes for 30 min, 3 h, and 15 min to remove lipids, dust, contaminants, and glue (i.e., for moths captured in automated traps). The samples were then air dried in a class‐100 fume hood and stored in glassine envelopes. Insect wings are often preferred for isotope analysis because they have limited tissue turnover (Lindroos et al., 2023). However, lepidopteran wings have very low sulfur content (<0.01% in spruce budworm moths) as they are primarily composed of chitin, (C_8_H_13_O_5_N)_*n*_ (Kaya et al., 2015). We analyzed sulfur isotopes using procedures similar to those for foliar samples (see *Foliar sulfur isoscape*), but smaller sample weights (2 ± 0.2 mg) with thoraces and heads sampled in priority and part of the abdomen incorporated for low‐mass samples. Since spruce budworms are not known to eat or drink as adults in the wild and are capital breeders (Rhainds, 2015), it is likely that individual δ^34^S values of the adult reflect those of the larvae in most tissues. We also regularly analyzed an internal matrix‐matched standard that we created from ground and homogenized spongy moth (*Lymantria dispar*, Linnaeus, 1758) heads and thoraces. This quality check standard gave δ^34^S = 2.7‰ ± 0.3‰ (*n* = 3), supporting an analytical precision of ±0.3‰.

#### Isoscape calibration to spruce budworm moth tissue

We generated a δ^34^S_moth_ isoscape using the *assignR* package (Ma et al., 2020). We used the *calRaster* function to fit a linear model relating δ^34^S_moth_ from the known‐origin dataset and the random forest δ^34^S_foliar_ isoscape. To evaluate the quality of the calibrated isoscape, we used the *QA* function and explored granularity (how concentrated assignments are), bias (systematic deviation from true origin), and the odds ratio of posterior probabilities of known‐origin locations relative to random ones.

### Automated trap collections and moth migration assessment

#### Trap collections and inferences of migration

We used automated pheromone traps (Trapview+ model, EFOS d.o.o., Slovenia) managed by the Canadian Forest Service and Provincial Governments to collect male spruce budworm across their distribution and expansion range (see Dargent et al., 2023 for details). These traps use sticky paper to collect specimens and take four photos daily (at approximately 05:00, 11:00, 19:00, and 23:00 h local time) to assess the approximate time of capture of each individual. Moths were qualitatively classified between locals and immigrants by combining phenology modeling, climate conditions at the trap location, abundance of capture from previous days, and time of capture (Greenbank et al., 1980; Sanders et al., 1978). Broadly, individuals captured in the evening/early night were generally classified as locals, whereas moths captured in the early morning or during a sudden capture peak were classified as immigrants. We selected specimens from two automated traps in Arisaig (45.75° N, −62.16° W) and Inverness (46.196° N, −61.294° W) in Nova Scotia from a known immigration event (i.e., July 22–23, 2020) to test δ^34^S as a pest dispersal‐tracing approach (Appendix S1: Figure S3). The immigration event at these sites had been documented through several independent lines of evidence, including: (1) a sudden peak of captures three to eight times higher than typical capture numbers, (2) low (<10 captures) moth activity in New Brunswick and surrounding areas on the night of immigration (Appendix S1: Figure S3), and (3) δ^2^H and ^87^Sr/^86^Sr values from captured individuals that strongly differed from those of locals captured in previous weeks (Dargent et al., 2023).

### Assignment of immigrant provenance

#### Continuous‐surface probabilistic assignment

We used the *pdRaste*r function in the *assignR* package (Ma et al., 2020) to generate continuous‐surface probabilistic maps of natal origin for all specimens sampled at capture sites between 2019 and 2020, including purported locals and immigrants. We then sequentially integrated the isotopic probabilistic assignments with HYSPLIT trajectories to refine origin estimates.

#### HYSPLIT atmospheric dispersal trajectories

We reconstructed the flight trajectory of potential immigrants captured in Arisaig and Inverness on the night of July 22–23, 2020, using HYSPLIT (v. 5.2.0), a Lagrangian atmospheric transport and diffusion model (Stein et al., 2015). Since spruce budworm's mass exodus behavior is density‐dependent (Morris, 1963; Régnière & Nealis, 2007), immigrants must have started from areas of high population density. To approximate population density, we combined and resampled spruce budworm defoliation maps for 2020 from the province of Quebec (Ministère des Ressources Naturelles et des Forêts, 2020) at 1‐km resolution. No spruce budworm defoliation was detected in Nova Scotia and the neighboring provinces of New Brunswick and Newfoundland in 2020, so defoliation was set to zero in these regions. We then performed dispersal simulations from all defoliated cells. Following Greenbank et al. (1980), these dispersal simulations incorporated a “moth velocity” parameter (i.e., 2.5 m/s) (e.g., Otuka et al., 2023) that accounts for active flying by moths. We generated plausible flight trajectories for the dates of July 21, 2020, and July 22, 2020, starting at 19:00, 21:00, and 23:00 h Atlantic Daylight Time (ADT), at 300‐, 600‐, and 900‐m altitude, for a duration of 9 h. We used meteorological data from the North American Mesoscale Forecast System at 12‐km resolution. We identified trajectories that landed inside or intersected a radius of 10 km around each trap, and evaluated temperature and altitude during the migratory flight trajectory to confirm that trajectories did not occur below the temperature flight threshold of spruce budworm (i.e., <15°C) or got close to ground level (i.e., <250 m), which could have triggered landing before reaching the traps.

### Statistical analyses

All statistical analyses were performed using R version 4.3.1 (R Core Team, 2020, http://www.r-project.org). R scripts and data are available in Dargent et al. (2026) at https://doi.org/10.17605/OSF.IO/MCQS9.

## RESULTS

### Foliar sulfur isoscape

**FIGURE 2:**
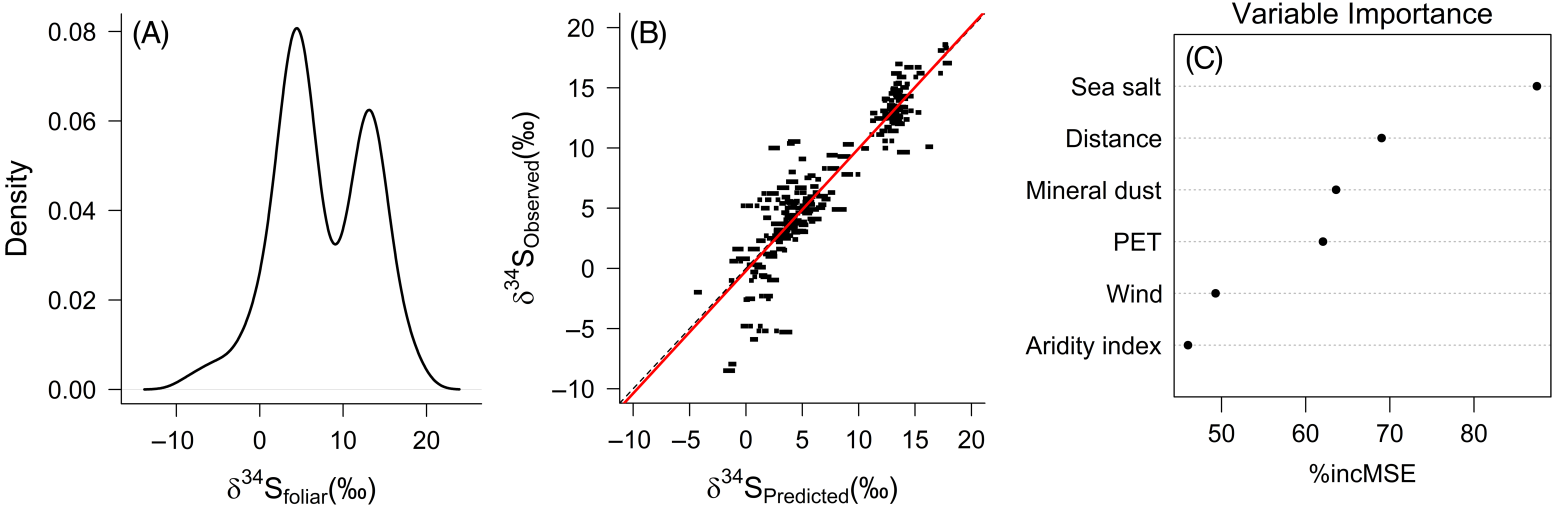
(A) Distribution of *δ* ^34^S_foliar_ values in spruce, balsam fir, and milkweed samples across North America. (B) Cross‐validation of predicted versus observed *δ* ^34^S_foliar_ values using random forest regression. The red line represents the best‐fit linear model and the broken line a 1:1 relationship between predicted and observed values (*R* ^2^ = 0.857; root mean squared error [i.e., RMSE] = 2.1‰). (C) Variable importance plots of *VSURF*‐selected predictors based on the mean decrease in accuracy, presented as the percent increase in mean squared error (%incMSE). PET, potential evapotranspiration.

The δ^34^S of the plant samples ranged from −8.5‰ to 18.6‰ (mean = 7.46‰) and displayed a bimodal distribution (Figure 2A). The random forest model identified six predictors of δ^34^S_foliar_ in North America, including sea salt aerosol deposition, distance to the coast, average mineral dust deposition, potential evapotranspiration, wind speed, and aridity index (i.e., mean annual precipitation divided by mean annual evapotranspiration) (Figure 2C). The model explained 85.7% of the variance in δ^34^S values and had an RMSE of 2.1‰, which is 7.6% of the range of values we collected (Figure 2A,B). The residuals of our model were normally distributed but tended to overpredict lower δ^34^S_foliar_ values, particularly for samples collected in the Northwest Territories (Figure 2B).

**FIGURE 3:**
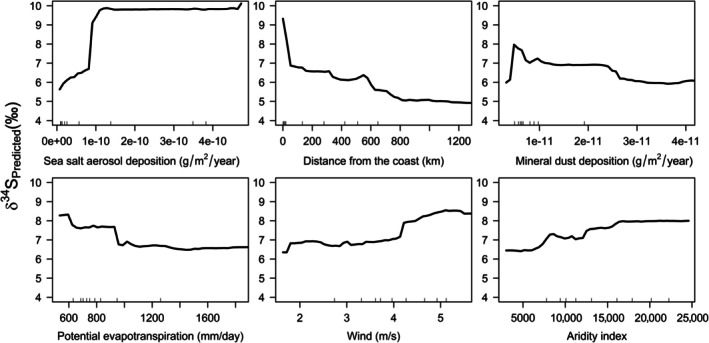
Partial dependence plots of *VSURF*‐selected predictors with predicted *δ* ^34^S_foliar_ values and the predictors.

Among the significant predictors, sea salt aerosol deposition, a proxy of oceanic sulfate deposition, showed a threshold relationship with δ^34^S_foliar_ values, with higher values near the coast, particularly in eastern Canada (Figure 3; Appendix S1: Figure S4A). Similarly, distance from the coast, aridity index, and potential evapotranspiration showed lower δ^34^S_foliar_ values away from the coast or in drier conditions. Wind velocity, which controls the distance and size of particles carried via atmospheric deposition (e.g., dry sea salt spray), showed a strong relationship with δ^34^S_foliar_ values, with higher δ^34^S_foliar_ within a narrow coastal band (Appendix S1: Figure S4B). Dust aerosol deposition also showed a threshold relationship with δ^34^S_foliar_ values, following a broad latitudinal gradient increasing northward (Appendix S1: Figure S4C,D).

**FIGURE 4:**
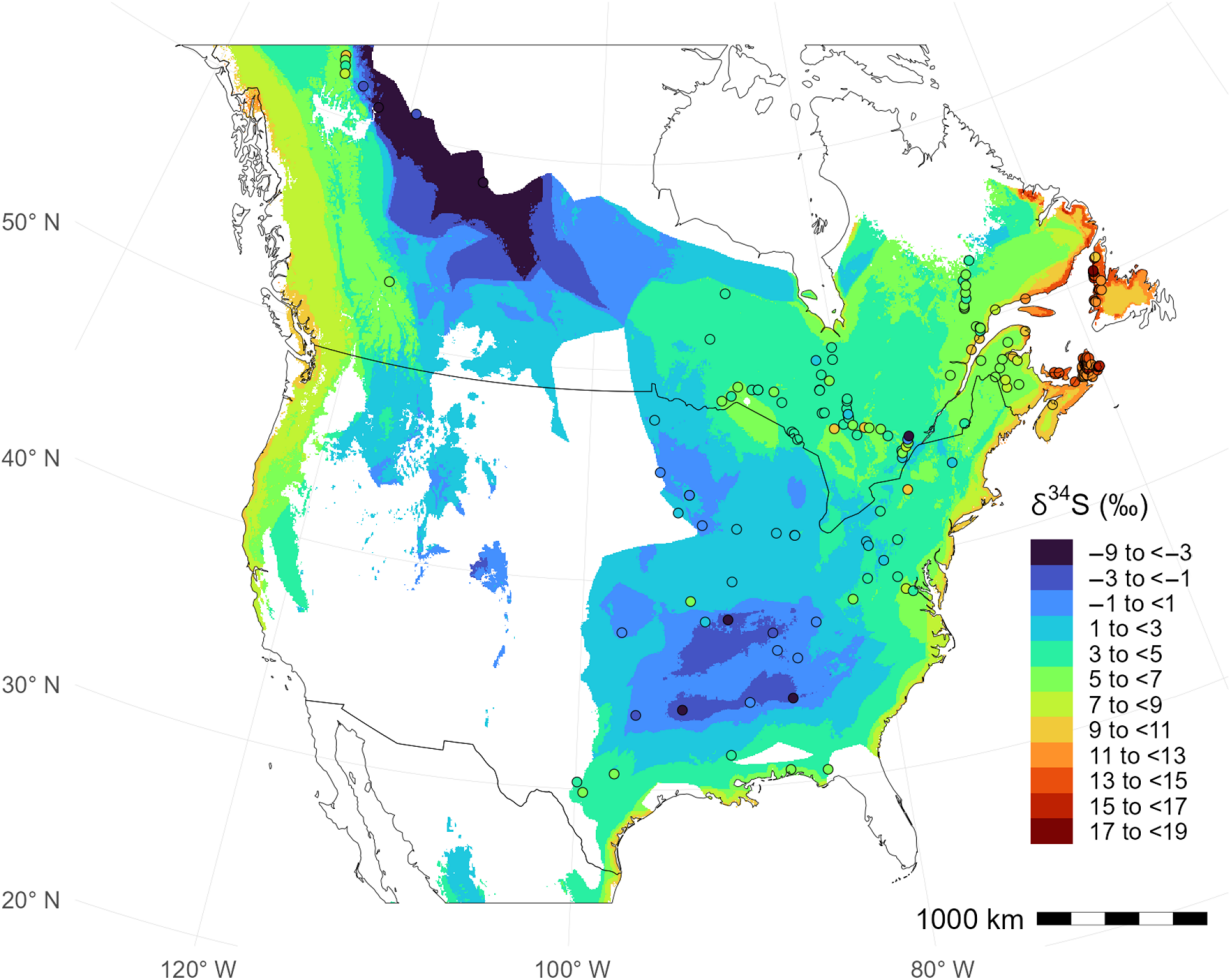
Predicted *δ* ^34^S_foliar_ from random forest regression across eastern North America. Points represent plant sampling locations colored according to the *δ* ^34^S_foliar_ color scale. Predictions are only provided for areas where predictors have values within the training set. Border polygons are from *rnaturalearth* (Massicotte & South, 2023). See Appendix S1: Figure S5 for the uncertainty layer.

Overall, our δ^34^S_foliar_ isoscape reveals a strong gradient of decreasing δ^34^S_foliar_ with increasing distance from coastal areas and very high δ^34^S_foliar_ values within a narrow coastal band influenced by oceanic winds (δ^34^S_foliar_ mean isoscape—Figure 4; δ^34^S_foliar_ uncertainty isoscape—Appendix S1: Figure S5). Away from the coast, δ^34^S_foliar_ values become more influenced by mineral dust deposition (Figure 4). Interestingly, despite the known influences of certain lithologies (e.g., black shales and evaporites) and anthropogenic pollution on ecosystem's δ^34^S values (e.g., Kabalika et al., 2020; Nehlich, 2015), the random forest model did not detect any direct influence of geological variables or human‐derived atmospheric deposition variables.

### Relationship between plants and moths in the known‐origin dataset

#### Calibration equation

**FIGURE 5:**
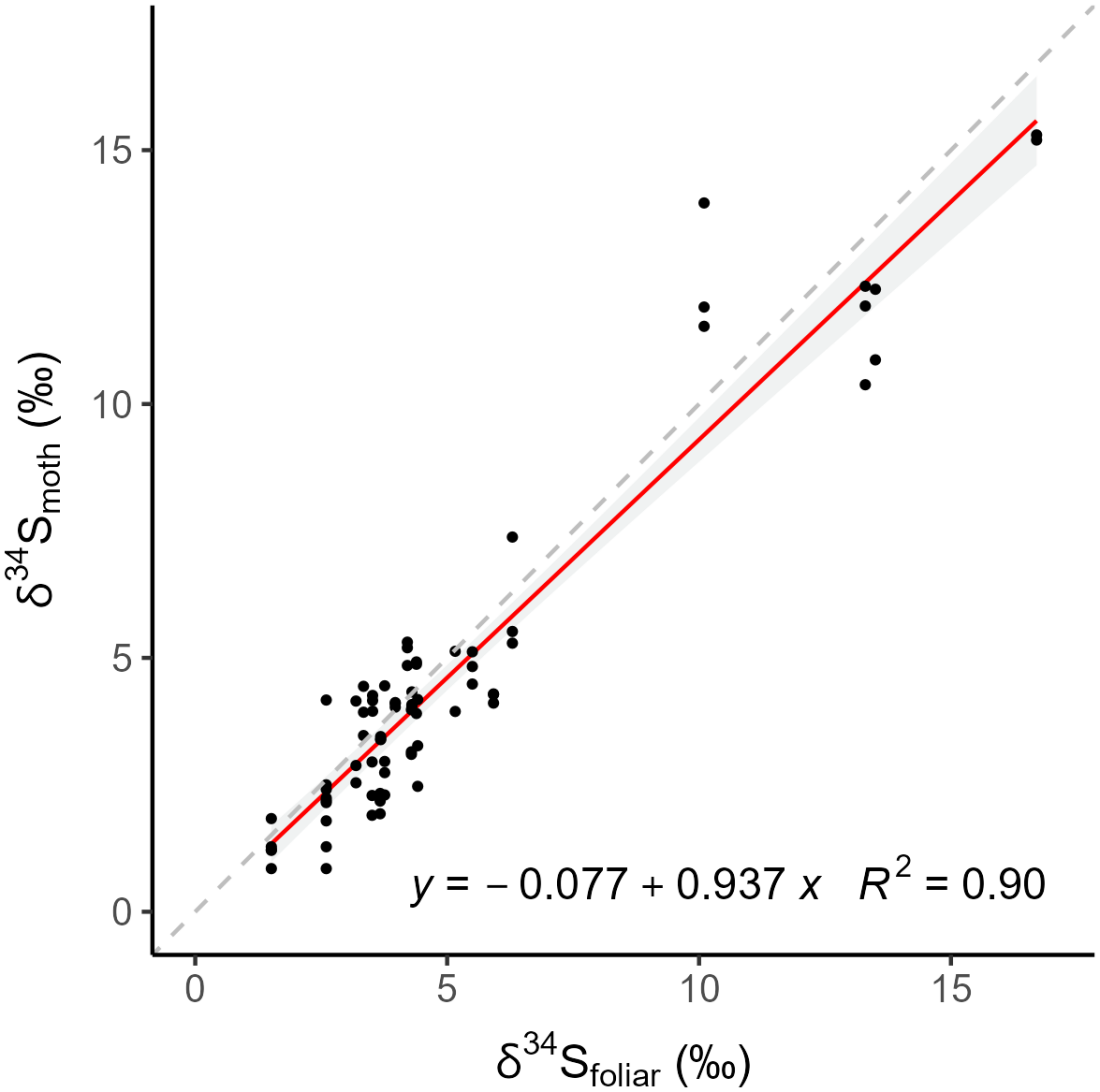
Relationship between individual moth *δ* ^34^S values (*δ* ^34^S_moth_; *n* = 75) and the average tree *δ* ^34^S value of their site of collection (*δ* ^34^S_foliar_; *n* = 23) was significant (*F*_1,73_ = 669.7, *p* < 0.001) and explained 90% of the variance in *δ* ^34^S_moth_ (*δ* ^34^S_moth_ = −0.08 + 0.94 × *δ* ^34^S_foliar_). The gray band represents the 95% CI and the broken line represents a 1:1 relationship between predicted and observed values.

We found a strong and significant relationship between δ^34^S_foliar_ and δ^34^S_moth_ values (*F*_1,73_ = 669.7, 𝑅^2^ = 0.90, *p* < 0.001, δ^34^S_moth_ = −0.08 + 0.94 δ^34^S_foliar_; see Figure 5), where moths have slightly lower *𝛿* ^34^S than the foliage they consume (i.e., −0.11‰ to 0.2‰ at the lower and higher ends of the foliar values we sampled, which is below our analytical error). An ANCOVA with known‐origin *𝛿* ^34^S_moth_ value as the response variable, *𝛿* ^34^S_foliar_ value as the covariate, and site nested within province as a factor showed that *𝛿* ^34^S_foliar_ was the strongest predictor (*F*_1,1_ = 1587, *p* < 0.001). Additionally, both province (*F*_1,1_ = 23, *p* < 0.001) and individual site within province (*F*_1,20_ = 5, *p* < 0.001) have minor but significant effects on *𝛿* ^34^S_moth_, possibly because these factors are correlated with local variation in δ^34^S values. Through the *calRaster* function of the *assignR* package, we obtained a strong correlation between δ^34^S_foliar_ isoscape and δ^34^S_moth_ values (δ^34^S_moth_ = −0.5 + 1.01 δ^34^S_foliar isoscape_, *R* ^2^ = 0.94) and a similar calibration equation to the δ^34^S_foliar_‐δ^34^S_moth_ regression. Differences between these two regressions are due to known‐origin moths collected at five sites without available foliage. Thus, we posit that δ^34^S_foliar_ values are a largely adequate proxy to develop insect δ^34^S isoscapes.

#### Intra‐site foliar δ ^34^S variation

We observed limited δ^34^S_foliar_ intra‐site variance associated with needle type and species. We found no difference between duplicates of the same needle tissue (21 independent trees). Duplicates showed an average δ^34^S_foliar_ difference of 0.16‰, well within the 0.3‰ analytical error. We found a small but nonetheless significant effect of foliage age on δ^34^S values (paired *t‐*test, *p* = 0.021, 𝑡_6_ = 3.11), with young needles having a 0.78‰ higher value than old needles on the few sites studied (*n* = 7). We found no significant effect of species when balsam fir and spruce δ^34^S_foliar_ were collected at the same site (paired *t‐*test, *p* = 0.83, 𝑡_8_ = 0.224). Intra‐site δ^34^S_foliar_ variance was also similar between spruce (0.37 ‰, range: 0.12‰–0.85‰) and fir (0.56‰, range: 0.19‰–1.02‰), and both are similar to the analytical error.

#### Intra‐site variation in moths' δ ^34^S at known‐origin sites

The average intra‐site δ^34^S_moth_ SD was 0.59‰ (range: 0.03‰–1.31‰). We used individual moth duplicates to test within‐moth variation, as a proxy for both within‐sample natural variation in δ^34^S values and analytical error. We found no significant difference between samples and their duplicates (*t*_12_ = 0.48, *p* = 0.64), with a mean difference of 0.11‰.

### Quality assessment of isoscapes and geographic assignment

While the δ^34^S_moth_ isoscape has strong geolocation potential, the model could be further improved, as the quality assessment analysis demonstrates some level of bias (i.e., on the 1:1 line; Appendix S1: Figure S6A—bias measure). This bias suggests that the δ^34^S_moth_ isoscape is too conservative, likely due to an overestimation of the uncertainty. This overestimation of uncertainty is a recurrent issue when using quantile random forest for estimating pseudo‐SD (Bataille et al., 2022), which we are actively trying to address. Despite being overly conservative, the δ^34^S isoscape can be used to effectively eliminate large portions of the study area as probable natal origin (Appendix S1: Figure S6B—granularity measure). The odds ratio plot indicates that known‐origin individuals are three times more likely to be assigned to their site of natal origin compared to random locations (Appendix S1: Figure S6C). These results demonstrate that sulfur isotopes are a viable method to trace the origin of insects, particularly for discriminating individuals from, or arriving at, coastal areas.

### Geographic assignments and intra‐site variations of local individuals

As a prerequisite to validate the application of δ^34^S geolocation, we generated geographic assignments of individuals captured in automated traps and classified as locals. Out of 32 individuals, 25 classified as locals through visual inspection of assignments (Arisaig *n* = 5 out of 6, Inverness *n* = 3 out of 3, St. Modeste *n* = 3 out of 3, Baldwin *n* = 3 out of 3, Zinc Mine rd. *n* = 3 out of 7, Forestville *n* = 2 out of 2, Gaspé *n* = 3 out of 3, Pikauba *n* = 2 out of 3, Petit Etang *n* = 1 out of 2) showed geographic assignments consistent with a local origin (Appendix S1: Figures S7–S15), whereas the remaining individuals showed very low probability of origin at the capture site. Additionally, captured locals have a significantly higher intra‐site δ^34^S_moth_ variance, with an average SD of 1.74‰ (range: 0.90‰–3.97‰), than those of known‐origin sites (*t*_8.99_ = −3.33, *p* = 0.009) (Appendix S1: Figure S16).

### Continuous‐surface geographic assignment and atmospheric dispersal of immigrant moths

**FIGURE 6:**
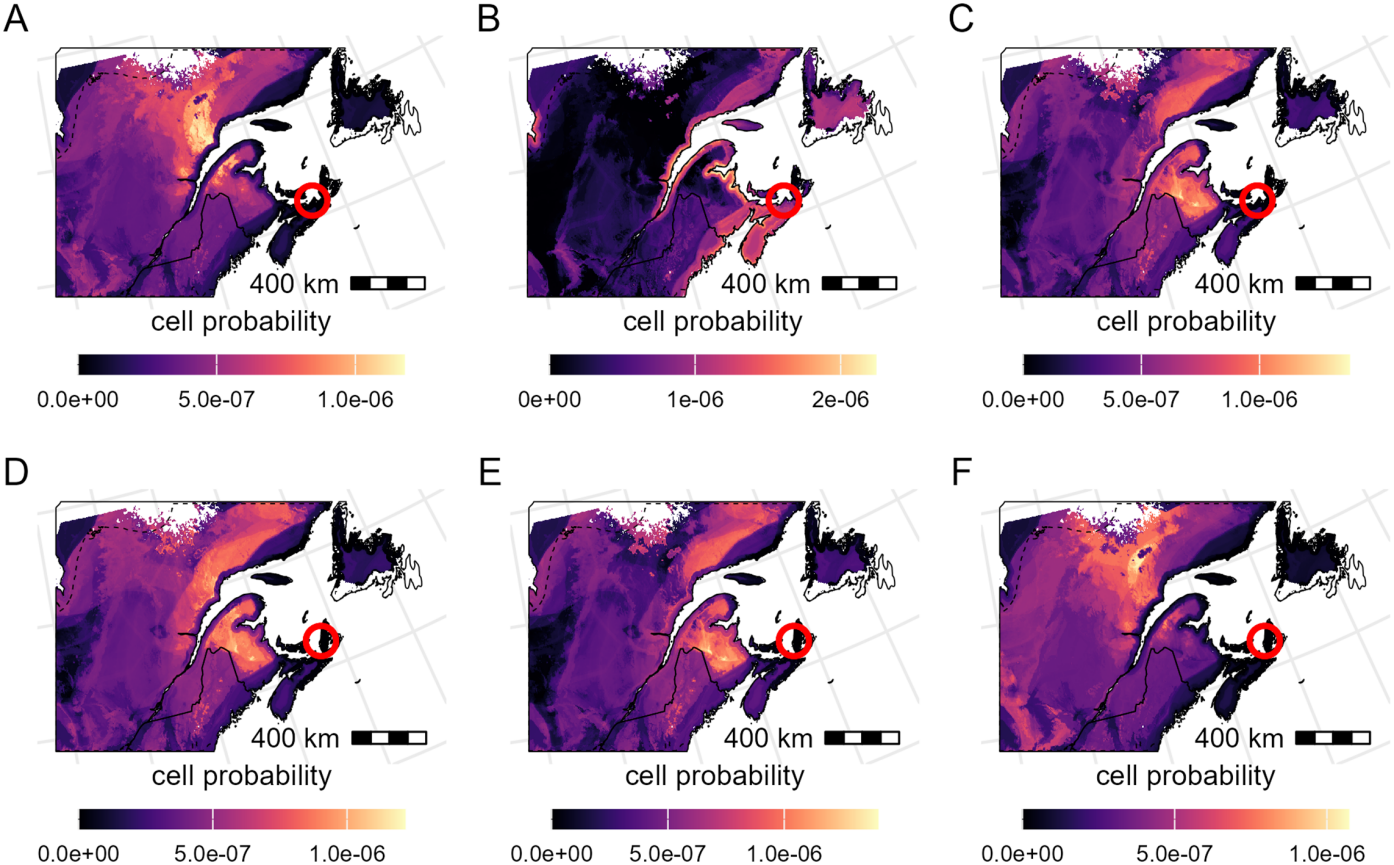
Posterior probability maps of each immigrant individual sampled from Arisaig (A–C) and Inverness (D–F). Each panel shows the probability of an individual having originated at each pixel given their *δ* ^34^S value and the pixel mean and uncertainty *δ* ^34^S_moth_ values. Trap locations are shown as red circles. The bright yellow areas represent sites with a higher probability of origin; black areas represent zero probability. The probability values of each map sum to 1, and values are only provided for areas within the spruce budworm distribution range (broken line), where all isoscape predictors have values within the training set (Figure 4). Border polygons are from *rnaturalearth* (Massicotte & South, 2023).

Moths from Arisaig (*n* = 3) and Inverness (*n* = 3) categorized as immigrants in the automated traps exhibited a large degree of δ^34^S_moth_ variation and generally lower values than those predicted by the δ^34^S_moth_ isoscape for these coastal locations in Nova Scotia (Figure 6), confirming that they likely did not originate from the capture location. High‐probability regions included locations in northern New Brunswick, the Gaspé Peninsula, and the north coast of the Saint Lawrence River (Figure 6; Appendix S1: Figure S17) and were highly distinct from those predicted for purported locals (Appendix S1: Figures S7, S8, and S18). Among these areas, only some parts of the Gaspé Peninsula and the north shore of the Saint Lawrence showed budworm defoliation in 2020, making these areas a more likely source of a mass migration event.

**FIGURE 7:**
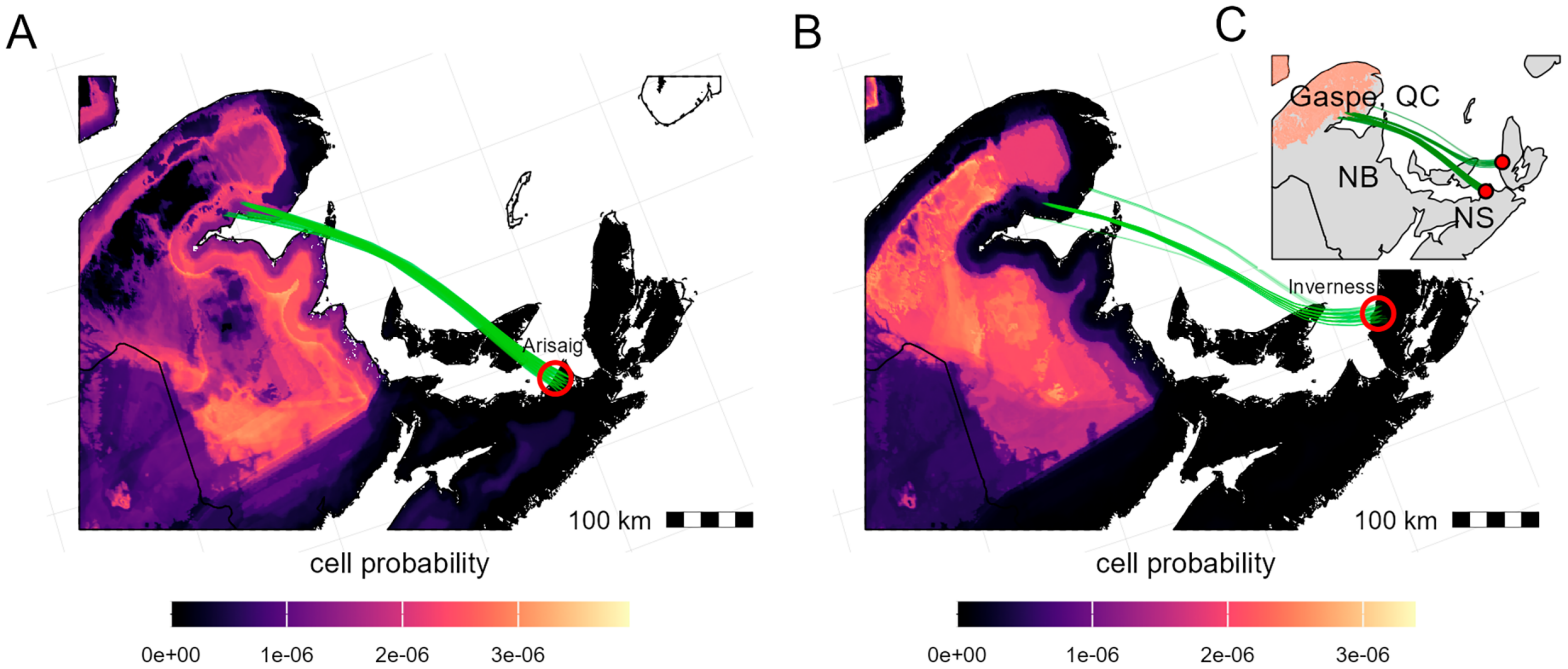
Joint posterior probability maps of all immigrant individuals sampled from Arisaig (A) and Inverness (B) with HYSPLIT trajectories (in green) from July 21, 2020, departing from the defoliated zone and landing within 10 km from the automated traps. Probability maps were evaluated for the extent of the study area, whereas initial HYSPLIT trajectories were constrained to defoliated areas (i.e., areas with high spruce budworm density). Trap locations are shown as red circles. Yellow areas represent sites of higher probability of origin; black areas represent zero probability of the individuals having originated there. The probability values of each map sum to 1, and values are only provided for areas within the spruce budworm distribution range, where all isoscape predictors have values within the training set (Figure 4). Border polygons are from *rnaturalearth* (Massicotte & South, 2023). (C) Map depicting areas that were defoliated in 2020 (orange) and HYSPLIT trajectories (green lines) to trap locations (red circles).

None of the simulated HYSPLIT trajectories within the approximate 6‐h window of the capture period (i.e., 17:00 h–23:00 h ADT) originated from areas of severe defoliation on the night of capture (July 22–23, 2020), indicating immigration from either earlier times and/or more distant areas. However, when reconstructing trajectories for the night before the capture event (July 21, 2020), 346 simulated trajectories originated from the outbreak zone on the southern part of the Gaspé Peninsula and landed less than 10 km from the Arisaig and Inverness traps (Figure 7). Of these, all trajectories remained above an altitude of 250 m, well above water and the tree canopy, whereas 11 trajectories were discarded because at one point before intersection the temperature fell below 15°C (i.e., below the temperature flight threshold of spruce budworm moths) (Appendix S1: Figure S19). The temperature and altitude along the remaining trajectories (20.5 > °C ≥ 15; 600 m > altitude > 250 m) are consistent with the dispersal ecology of the moth and would not have imposed physiological constraints or barriers to movement (Sanders et al., 1978). These remaining trajectories represent the most likely routes of the migrating eastern spruce budworm moths and include a water‐crossing of over 400 km. The δ^34^S‐based geographic assignment, defoliation maps, and successful HYSPLIT trajectories overlap in the southeastern Gaspé Peninsula, making this area the most likely natal origin of the moths that immigrated to Arisaig and Inverness on July 22–23, 2020.

## DISCUSSION

### Sulfur isoscape

The isoscape displays a progressive decline in δ^34^S_foliar_ values when moving away from the ocean (Figure 4), supporting previous studies about the dominance of marine sulfate deposition in controlling the continental δ^34^S variations in plants (Sparks et al., 2019; Tarrant & Richards, 2024), birds (Brlík et al., 2023; Hebert & Wassenaar, 2005), and mammals (Bataille et al., 2021, 2022; Zazzo et al., 2011), and consistent with patterns of δ^34^S variation in North America (Bataille et al., 2022; Bataille, Chartrand, et al., 2020; Valenzuela et al., 2011). Partial dependence plots show that sea salt aerosol deposition rates, distance to the coast, wind, aridity index, and potential evapotranspiration are important predictors of δ^34^S_foliar_ values (Figure 3). Interestingly, the relationship between δ^34^S_foliar_ and those predictors is not linear, with a sharp decline in the first few kilometers away from the coast, a plateau between 10 and 100 km from the coast (or when ecosystems become drier and less windy), followed by a more gradual decline extending to more than 600 km (Figure 3), supporting similar spatial gradients in Trinidadian δ^34^S_foliar_ (Sparks et al., 2019) and European δ^34^S_vertebrate_ isoscapes (Bataille et al., 2021). Wind and mean annual precipitation (represented by the aridity index and potential evapotranspiration) display a plateau where wetter and windier areas have higher δ^34^S_foliar_ values, likely reflecting the typical wet and windy conditions within a 100‐km band from the coast (Appendix S1: Figure S4). The low δ^34^S_foliar_ values away from the coast likely also reflect the increasing contribution of terrestrial or anthropogenic sources with low δ^34^S in continental precipitation. These δ^34^S_foliar_ patterns are promising to discriminate within a gradient of coastal to continental migratory insects.

Deposition rates of mineral dust are also an important predictor of δ^34^S_foliar_, likely because mineral dust particles scavenge sulfates from the atmosphere and contribute to increasing the sulfate deposition rate on ecosystems (Dupart et al., 2012). Consequently, areas with higher dust deposition have lower δ^34^S_foliar_ values because of the addition of terrestrial and anthropogenic sulfates with low δ^34^S to the ecosystems. For example, in the United States, the regions of high dust deposition surrounding the Midwest and southern United States, which have the highest rates of sulfur dioxide emissions, all show relatively low δ^34^S_foliar_ (Appendix S1: Figure S4). In summary, the δ^34^S_foliar_ isoscape reveals a predictable and broad range of spatial variation across our study area, which can be leveraged for geolocation, specifically to distinguish organisms that have metabolized local resources into their tissues at varying distances from the coast.

### δ ^34^S as a discrimination isotope for spruce budworm natal origins

The strong and nearly 1:1 relationship between δ^34^S_foliar_ and δ^34^S_moths_ indicates negligible or minimal fractionation of sulfur isotopes during trophic transmission for this folivorous insect, supporting previous findings for other animals (though some species show some fractionation; Raoult et al., 2024). Additionally, the limited variability between plant species and needle age also supports relatively homogeneous δ^34^S values within ecosystems. This conservation of δ^34^S values within tissues, species, and across trophic levels highlights the dominance of spatial processes in controlling sulfur isotope patterns and makes sulfur isotopes attractive for isotope geolocation relative to other elements that are strongly influenced by trophic and metabolic processes (e.g., hydrogen, carbon, or nitrogen).

We found low but non‐negligible amounts of variation among individual known‐origin moths from the same site (median SD = 0.59‰), whereas putative local moths showed significantly higher δ^34^S_moth_ variation (mean SD = 1.74‰), particularly at sites close to the ocean. This high intra‐site δ^34^S variability for purported local moths mirrors the high variability of hydrogen and strontium isotopes at those sites, further supporting significant local mobility of spruce budworm moths beyond what had been reported in the literature before the use of isotope tracers (Dargent et al., 2023). These observations suggest that “local” moths could move well beyond the few hundred meters reported in early studies by Sanders (1983) and/or that existing qualitative classification based on time of capture is not always effective at identifying migrants. The extent of this local movement ought to be explored further, as it informs the extent to which outbreaks can expand passively and without long‐distance dispersal.

### A new framework to trace insect pests

Single‐tool approaches to tracing pest movement have limitations, but by combining independent approaches we can improve the accuracy and precision of tracing pest immigration events. Arisaig and Inverness immigrants captured on the night of July 23, 2020, showed areas of high probability of natal origin around the Gaspé Peninsula, the northern shore of the Saint Lawrence, and New Brunswick (Figure 7). The δ^34^S‐derived estimates of natal origin exclude large parts of the study area. Some of those regions of high probability coincide with regions that were defoliated in 2020, making them strong candidates for dispersal source areas supported by independent ecological evidence associated with spruce budworm migratory patterns (i.e., the budworm density–defoliation patterns and population density–emigration behavior; Régnière & Nealis, 2019). Interestingly, the most probable areas of origin obtained from wind dispersal trajectories coincided with areas of high probability of natal origin based on δ^34^S_moth_ values. Additionally, the absence of local moth captures at Arisaig and Inverness on the days preceding the mass capture, combined with the lack of evidence for high larval densities indicative of high population levels in the vicinity of the traps (nor even in New Brunswick), further supports the nonlocal origin of these individuals. The most parsimonious explanation to integrate these results is that those immigrant moths found in Arisaig and Inverness dispersed on the previous night (i.e., July 21) from the Gaspé Peninsula. Together, isotope geolocation, defoliation maps, and atmospheric dispersal models, along with the network of automated pheromone traps with sensors to determine capture times, provide an unprecedented and high spatiotemporal resolution view of the ecology and migration characteristics of this major insect pest.

In this case study, we demonstrated that δ^34^S values are particularly useful to identify and track the migration of insects originating from areas located at different distances from the coast, where we see the steepest δ^34^S variations. However, the accuracy of these estimates of origin could be further improved by more extensive sampling of the study region, particularly in locations with δ^34^S values between 7‰ and 12‰ (Figure 2A), and by improvements to estimating the spatial uncertainty of the isoscape using quantile random forest (Bataille et al., 2022). While the precision of the δ^34^S geolocator will be strongest to track insect migration in this type of environmental setting, a key strength of isotope geolocation is the possibility to combine multiple isotopes. Using hydrogen and strontium isotopes along with sulfur can considerably increase the precision of geolocation (Bataille et al., 2021; Dargent et al., 2023), enabling the tracking of insect pests at fine spatiotemporal resolution in a range of environmental conditions.

### Management implications

Our new integrated tracking framework advances the possibility of sustainably managing insect pests. Knowing whether immigration events occur at a subregional (e.g., tens of kilometers) or regional (e.g., hundreds of kilometers) scale can help assess the required scale of coordination between actors involved in pest management. For example, for spruce budworm, subregional immigrations are more likely to concern provincial or local jurisdiction management strategies, whereas regional, long‐distance, immigration would require higher cooperation among provinces to effectively manage outbreaks. So far, Canadian government scientists have informed management of spruce budworm moth outbreaks through the Spruce Budworm Early Intervention Strategy (e.g., Johns et al., 2019), which uses BTK (i.e., a bacterium‐derived toxin that targets Lepidoptera larvae) to spray areas of rapidly growing populations and keep them below outbreak levels. Our study demonstrates that eastern spruce budworm moths can migrate hundreds of kilometers across provincial borders. Thus, spruce budworm outbreak management would benefit from inter‐provincial scale cooperation and coordination, as outbreaks in one province can spread to others through migration. This cooperation could involve a systematic analysis of individuals captured by pheromone traps in previous years and atmospheric dispersal models to identify common inter‐provincial migratory corridors and assess the risk of future inter‐provincial dispersal. This would support the management of high‐density areas not only at the local level but also as a key risk for generating long‐distance migrants to other vulnerable provinces from that site (e.g., downwind). The coordination for these activities could include interjurisdictional agreements, such as those previously established for mountain pine beetle (*Dendroctonus ponderosae*) between the provinces of Saskatchewan and Alberta (Hodge et al., 2017), in which one jurisdiction would manage an area of high population density because it may generate migrants that would likely spread to another jurisdiction.

The integrated framework we propose is transferable to evaluate, at high temporal and spatial resolution, the migration ecology of many forest and agricultural insect pests, as well as migratory insects of conservation interest. Combining automated pheromone traps with isotopic analyses and atmospheric dispersal models enables the precise identification of immigrants and their origins, and offers the possibility to study the biotic, climatic, meteorological, and environmental controls of outbreak spread. Such knowledge could be leveraged along with high‐spatiotemporal‐resolution remote sensing of population data to predict, in nearly real time, the population dynamics and outbreak spread risks of a given insect pest. Using these quantitative monitoring and forecasting tools, researchers could considerably improve the management or conservation strategies of migratory insects in both present and future climate scenarios.

## CONCLUSION

Our study introduces a novel, integrated framework that combines automated traps equipped with sensors, isotopic analysis (i.e., δ^34^S geolocation), field surveys of defoliation representative of ecological dynamics (i.e., population density and mass exodus triggers), and atmospheric dispersal modeling with physiological constraints (i.e., inclusion of insect flight velocity, temperature limits on flight, and altitude) to effectively trace the migration of insect pests. This framework provides the possibility to track the origin and routes of insect migration at high spatiotemporal resolution, information that is crucial for understanding the ecological conditions triggering outbreak spread. By enabling precise identification of immigrant origins and routes, this novel framework provides crucial data for enhancing cooperation between management and stakeholders and for making management strategies targeted, scalable, and sustainable. While this framework was demonstrated on spruce budworm, it is transferable and promises significant advancements in the sustainable management of various forest and agricultural insect pests, particularly those dispersing at the adult stage over long distances (>100 km) with limited feeding during and after migration (e.g., Asian spongy moth [*Lymantria dispar asiatica*], mountain pine beetle [*D. ponderosae*], fall armyworm [*Spodoptera frugiperda*]). The framework's high modularity makes it applicable to various regions, for pests with different ecologies, and can be combined with other innovations such as artificial intelligence (AI)‐based identification, radar tracking, and high‐resolution remote sensing to improve its spatial resolution. The combination of automated trap monitoring, wind trajectory modeling, and next‐generation isotope geolocation goes beyond insect pest management and also applies to other migratory insects of conservation or bio‐surveillance interest. Developing such integrated monitoring networks across the globe is a promising avenue to evaluate and manage the global risks and benefits of migratory insects for ecosystems, food production, and human societies in the face of global change.

## AUTHOR CONTRIBUTIONS

Felipe Dargent, Jean‐Noël Candau, and Clement P. Bataille: Conceived and designed the project. Felipe Dargent, Jean‐Noël Candau, Kerry Perrault, and Megan S. Reich: Collected data. Felipe Dargent, Marrissa Miller, Nilofar Benvidi, and Joshua Aibueku: Prepared and analyzed samples. Felipe Dargent, Jean‐Noël Candau, Kala Studens, Megan S. Reich, Marrissa Miller, and Clement P. Bataille: Analyzed the data. Felipe Dargent, Jean‐Noël Candau and Clement P. Bataille: Contributed reagents; materials; and analysis tools. Felipe Dargent: Wrote the first draft and revised it with input from coauthors. Felipe Dargent, Megan S. Reich, Jean‐Noël Candau, and Clement P. Bataille: Revised and edited the manuscript. All authors reviewed and contributed to the article and approved the submitted version.

## FUNDING INFORMATION

This study was funded through an Early Intervention Strategy against Spruce Budworm Phase III awarded to Felipe Dargent and Jean‐Noël Candau and an Early Intervention Strategy against Spruce Budworm Phase III Small Scale Research Program awarded to Clement P. Bataille. The project was also covered by the Healthy Forest Partnership Early Intervention Strategy against Spruce Budworm Phase II Contribution Program awarded to the Invasive Species Centre by Natural Resources Canada ‐ Canadian Forest Service ‐ Pest Risk Management and contracted to Clement P. Bataille. Clement P. Bataille also received funding from the Natural Sciences and Engineering Research Council of Canada (NSERC) Discovery Grant RGPIN‐2019‐05709.

## CONFLICT OF INTEREST STATEMENT

The authors declare no conflicts of interest.

## Supporting information


Appendix S1.

## Data Availability

Data and code (Dargent et al., 2026) are available in the Open Science Framework at https://doi.org/10.17605/OSF.IO/MCQS9.
