# Supplementary material for: An integrated framework to identify and characterize regional‐scale insect dispersal
Source: Ecol Appl. 2026 Apr 20;36:e70230. doi: 10.1002/eap.70230 (PMC13095504; doi:10.1002/eap.70230)
Supplement: Supplementary file 1 — Appendix S1. [file EAP-36-e70230-s001.pdf]

Appendix S1

An integrated framework to identify and characterize regional-scale insect dispersal

Felipe Dargent, Megan S. Reich, Marrissa Miller, Kala Studens, Nilofar Benvidi, Kerry Perrault, Joshua Aibueku, Brent Holmes, Clement P. Bataille, Jean-Noël Candau

Ecological Applications

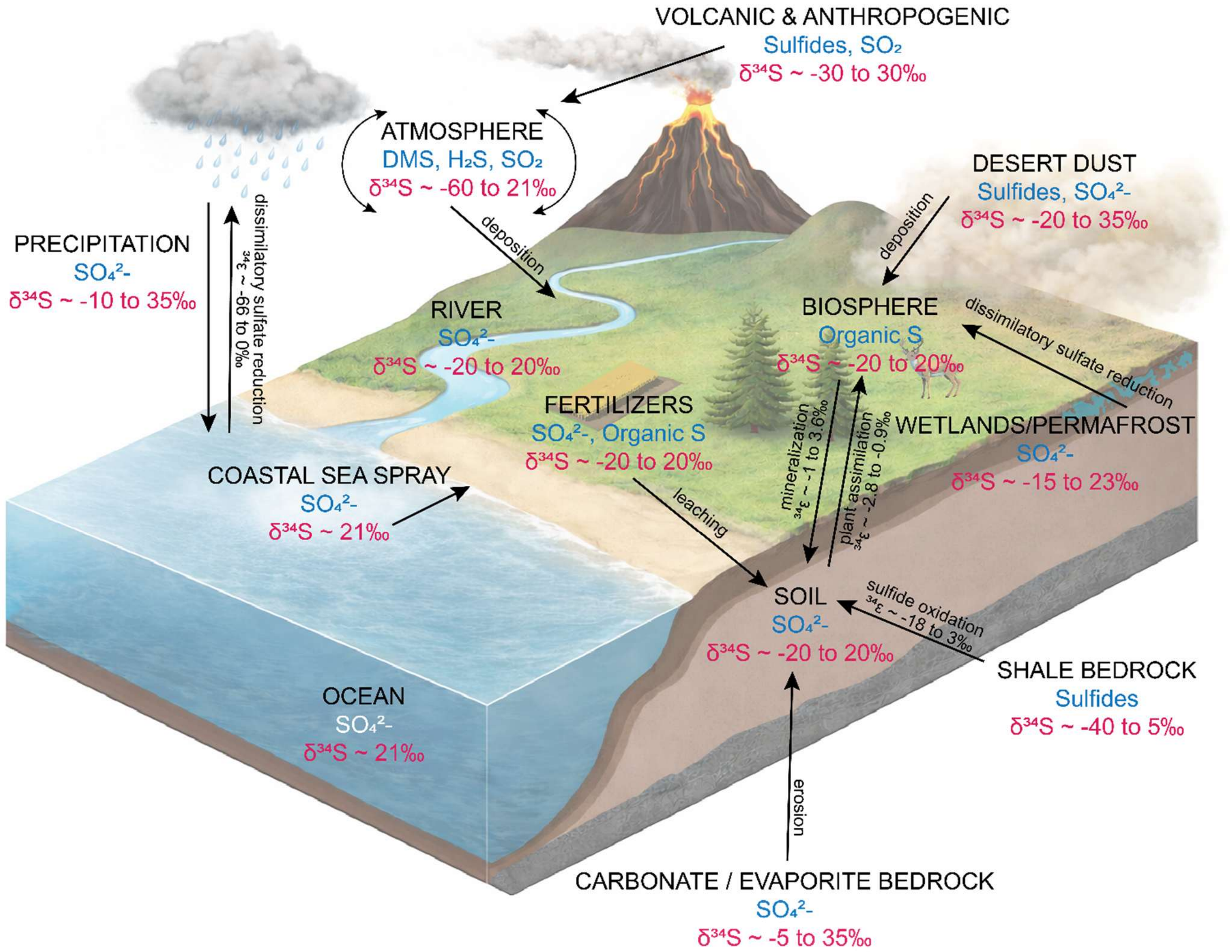

Figure S1: Overview of the sulfur cycle and expected δ^{34}S values in different environmental components

Sulfur isotopes (referred to as δ^{34}S) vary on the landscape with atmospheric deposition processes or geology and have shown promising potential for geolocation in archeology (Bataille et al., 2021) and bird ecology (Brlík et al., 2024; Brlík et al., 2023). Newton (2021) demonstrated that moths collected across the UK showed consistent spatial patterns with δ^{34}S gradient from the coast to inland regions. However, δ^{34}S has not been tested as a geolocator for insect migration. Sulfur isotopes are attractive as an insect geolocator because sulfur is a macronutrient present in measurable amounts in many insect tissues through two protein-forming amino acids, the essential methionine and the non-essential cysteine (Tcherkez & Tea, 2013). The sulfur isotope cycle is also well-characterized, primary producers (i.e., plants) obtain sulfur primarily from soils and more rarely through atmospheric uptake as sulfur oxides and carbonyl sulfides (Tcherkez & Tea, 2013; Trust & Fry, 1992). In ecosystems close to the coast, soil sulfur is derived from

atmospheric deposition of marine sulfates deposited either by short-distance dry deposition of sea spray or wet deposition of sulfates dissolved in precipitation (Figure 1). The modern ocean sulfates have homogeneous and elevated $\delta^{34}S$ +21 ± 0.2 ‰ (Böttcher, Brumsack, & Dürselen, 2007). Coastal ecosystems with high rates of marine sulfate inputs have usually higher $\delta^{34}S$ values than those located more inland (Bataille et al., 2021; Sparks et al., 2019). Conversely, in ecosystems with low marine sulfate deposition, other processes often dominate sulfur cycling including sulfates from sulfide oxidation from soil minerals (e.g., black shales) with very low $\delta^{34}S$ values (<0 ‰), weathering of sulfate-rich rocks (e.g., evaporites) with high but variable $\delta^{34}S$ values, atmospheric deposition of sulfates from human emissions with generally low $\delta^{34}S$ values or dust deposition of sulfur-containing minerals from desertic areas or volcanic ash with lower $\delta^{34}S$ values (Nehlich, 2015), and sulfide accumulation from bacteria and archaea activities in wetlands and under thawing permafrost with low sulfude and higher sulfate $\delta^{34}S$ values (Stevens et al., 2025; Jones et al. 2020; Orem et al. 2011; Harrison & Thode, 1958). The complex cycling and multiple isotopically-distinct sources of sulfur in ecosystems might lead to isotope patterns independent from hydrogen and strontium providing a new tool for high-resolution insect tracing. Figure base images created using generative AI.

Table S1: List of climatic, geological, soil and atmospheric deposition variables used in the variable selection process.

Variable	Description	Resolution	Source
Climate			
r.mat	Mean annual temperature (°C)	30 arc-sec	Harris et al., 2020
r.map	Mean annual precipitation (mm/year)	30 arc-sec	Harris et al., 2020
r.ai	Global aridity index	30 arc-sec	Trabuco & Zomer 2019
r.pet	Global potential evapo-transpiration (mm/year)	30 arc-sec	Trabuco & Zomer 2019
r.wind	Wind speed (m/s, 1970-2000)	30 arc-sec	Fick & Hijmans 2017
r.vapor	Water vapour pressure (kPa, 1970-2000)	30 arc-sec	Fick & Hijmans 2017
r.tmin	Mean annual minimum temperature (°C, 1970-2000)	30 arc-sec	Fick & Hijmans 2017
r.tmax	Mean annual maximum temperature (°C, 1970-2000)	30 arc-sec	Fick & Hijmans 2017
r.solar	Solar radiation (kJ/m^2/day, 1970-2000)	30 arc-sec	Fick & Hijmans 2017
Geology			
r.m1	Global median bedrock model ($^{87}Sr/^{86}Sr$ ratio)	1 km	Bataille et al., 2018
r.srsrq1	Global 1st quartile bedrock model ($^{87}Sr/^{86}Sr$ ratio)	1 km	Bataille et al., 2018
r.srsrq3	Global 3rd quartile bedrock model ($^{87}Sr/^{86}Sr$ ratio)	1 km	Bataille et al., 2018
r.age	Global mean terrane (basement) age attribute in millions of years (Ma)	1 km	Mooney et al., 1998
r.minage_geol	Minimum geological age attribute from GLiM (Ma)	1 km	Hartmann & Moosdorf 2012, Bataille et al., 2020
r.maxage_geol	Maximum geological age attribute from GLiM (Ma)	1km	Hartmann & Moosdorf 2012, Bataille et al., 2020
r.meanage_geol	Mean geological age attribute from GLiM (Ma)	1 km	Hartmann & Moosdorf 2012, Bataille et al., 2020
r.GUM	Global unconsolidated materials (GUM) type	1 km	Börker et al., 2018
r.bouger	Bouger gravity anomaly (mGal)	2 arc-min	Balmino et al., 2012
r.elevation	SRTM elevation model (m)	90 m	Jarvis et al., 2008
Soil Properties			
r.clay	Soil clay fraction weight %,) in the topsoil (5-15 cm depth)	250 m	Poggio et al., 2021
r.ph	Soil pH in H_2O solution x10	250 m	Poggio et al., 2021
r.cec	Cation exchange capacity (cmol(+)/kg)	250 m	Poggio et al., 2021
r.bulk	Bulk density of fine earth fraction (kg/m^3)	250 m	Poggio et al., 2021
r.ocs	Soil organic carbon stock (kg/m^2, 0-30 cm depth)	250 m	Poggio et al., 2021
r.w0010	Soil water content at -10 kPa (available water, 5-15 cm depth)	250 m	Poggio et al., 2021
r.w0033	Soil water content at -33 kPa (field capacity, 5-15 cm depth)	250 m	Poggio et al., 2021
r.w1500	Soil water content at -1500 kPa (wilting point, 5-15 cm depth)	250 m	Poggio et al., 2021
r.w0010_30	Soil water content at -10 kPa integrated to 30 cm depth	250 m	Poggio et al., 2021
r.soc	Soil organic carbon content (g/kg weight, 5-15 cm depth)	250 m	Poggio et al., 2021
r.ocd	Soil organic carbon density (kg/m^3, 5-15 cm depth)	250 m	Poggio et al., 2021
r.nitrogen	Soil nitrogen content (g/kg, 5-15 cm depth)	250 m	Poggio et al., 2021
r.perma	Permafrost cover probability	1 km	Obu et al., 2018
r.magt	Mean annual ground temperature (°C)	1 km	Obu et al., 2018
r.sw	Soil moisture (m^3/m^3, 0-5 cm depth)	15 km	Guevara et al., 2020
r.wetland	Global wetland/ inundation extent (fractional cover, 0-1)	15 arc-sec	Lehner & Döll 2004

Atmospheric Deposition			
r.dust	Global dust deposition rate ($g/m^2/year$)	1°x1°	Chien et al., 2016
r.salt	Global sea salt aerosol deposition ($g/m^2/yr$)	1°x1°	Chien et al., 2016
r.distance	Distance to the coast (km)	30 arc-sec	Bataille et al., 2021
r.volc	Volcanic deposition rate ($kg/m^2/s$)	0.5°	Brahney et al., 2015
r.fire	Black carbon deposition from wildfires ($kg/m^2/s$)	1°x1°	Chien et al., 2016
r.foss	Fossil fuel black carbon deposition ($kg/m^2/s$)	1°x1°	Chien et al., 2016

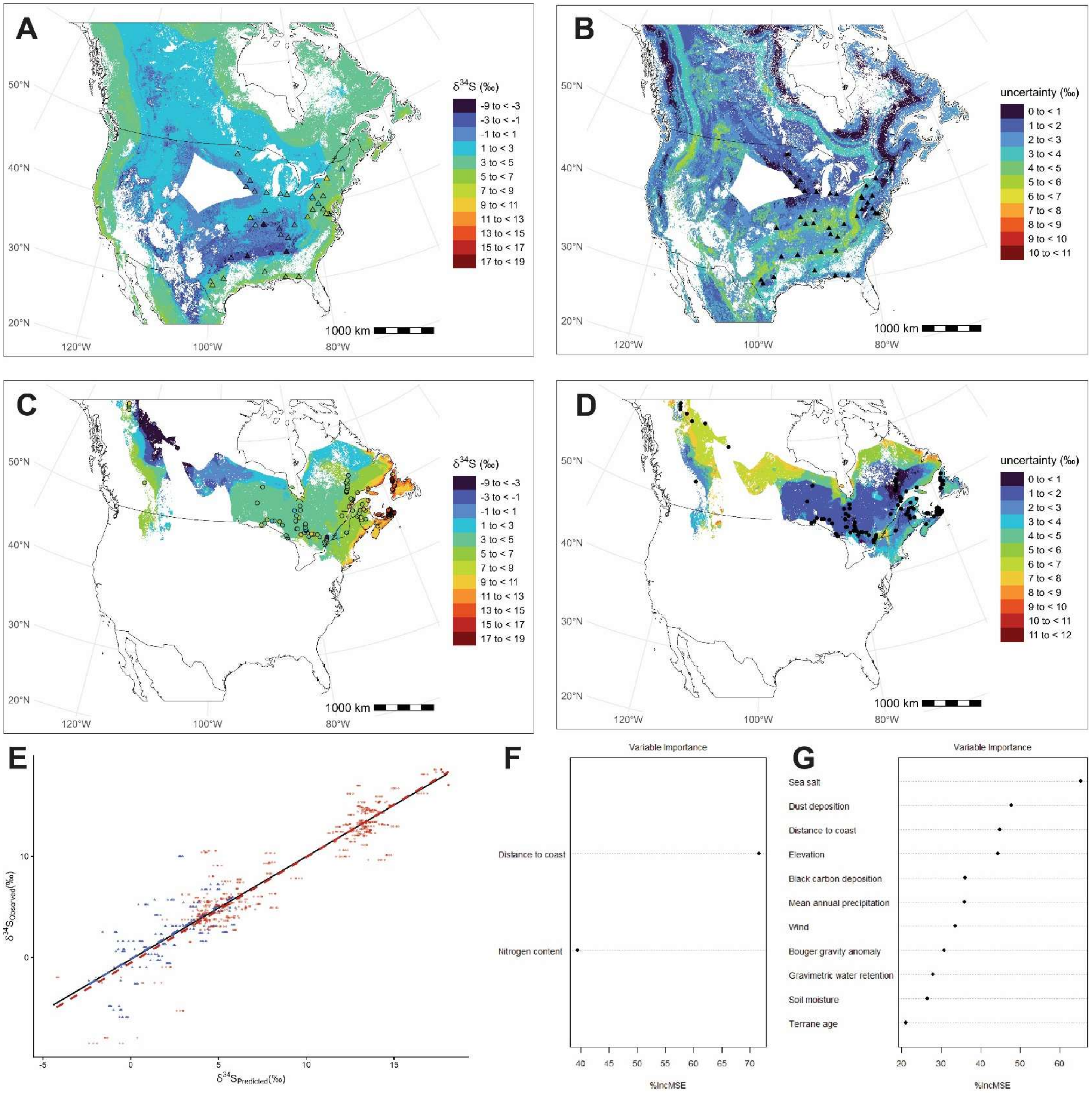

Figure S2: Foliar sulfur isoscapes of North America modelled using random forest regression trained with either milkweed (A, B, F) or spruce and balsam fir (C, D, G). Predictions and uncertainty are only provided for areas where predictors have values within the training set. Border polygons are from *rnaturalearth* (Massicotte and South 2023). (A) Predicted mean $\delta^{34}S_{milkweed}$ of North America (RMSE = 2.29; R^2 = 0.63). Triangles represent milkweed

sampling locations (n = 41) colored according to the color scale of Figure 2. (B) Spatially explicit uncertainty from the random forest prediction for milkweed (maximum = 6.2‰). The uncertainty represents one standard deviation and was obtained from quantile random forest modeling (see Methods). Black triangles indicate locations where milkweed samples were collected. (C) Predicted mean $\delta^{34}S_{tree}$ of North America (RMSE = 1.83; R^2 = 0.89). Points represent tree sampling locations (n = 156) colored according to the color scale of Figure 2. (D) Spatially explicit uncertainty from the random forest prediction for balsam fir and spruce (maximum = 11.9‰). Black circles indicate locations where tree samples were collected. (E) Cross-validation of predicted vs. observed $\delta^{34}S_{milkweed}$ (blue triangles) and $\delta^{34}S_{trees}$ (red circles) using random forest regression. The lines represent the best-fit linear models (blue dashed line: milkweed; red dashed line: trees; black solid line: model from complete dataset from Figure 2). (F) Variable importance plot of VSURF-selected predictors for the milkweed sulfur isoscape based on mean decrease in accuracy. (G) Variable importance plot for the tree sulfur isoscape based on mean decrease in accuracy.

Table S2: Known-origin moths summary. Field reared moths used to calibrate $\delta^{34}S_{foliar}$ isoscape to $\delta^{34}S_{moth}$ isoscape. At each site we report the number of moths sampled, their average $\delta^{34}S_{moth}$ value whether foliage at the site was also available, and the location of collection.

Site	Province	n	Mean $\delta^{34}S_{moth}$	Foliar site match	Latitude	Longitude
Bl101E	NL	3	12.47	Y	50.87956	-56.91763
Bl305	NL	3	12.97	Y	49.25215	-57.87666
Bl322	NL	3	11.54	Y	49.39097	-57.66103
Bl405	NL	2	11.57	Y	48.93961	-58.40058
C12	NL	2	15.25	Y	50.11000	-57.66000
C6	NL	3	14.97	Y	49.43474	-57.86359
Block 1	NB	1	7.70	N	47.34988	-66.38909
Block 10	NB	1	7.40	N	47.56949	-66.51317
Block 5	NB	1	5.70	N	47.76837	-66.32836
DO3	ON	5	1.29	Y	47.05850	-79.78170
DR1	ON	3	3.19	Y	46.95170	-79.77200
G2	ON	3	4.57	Y	46.63493	-80.24853
KO1	ON	3	3.95	Y	47.31834	-81.76209
KO11	ON	9	2.18	Y	47.07022	-79.76510
KO13	ON	2	4.54	Y	46.52517	-79.55433
KO14	ON	3	4.08	Y	46.03226	-79.36216
KO16	ON	3	4.81	Y	46.25990	-78.34390
KO2	ON	3	2.38	Y	47.31834	-81.58175
KO3	ON	3	4.15	Y	48.43153	-81.68478
KO4	ON	3	5.12	Y	48.40571	-81.68267
KO5	ON	3	4.12	Y	49.12400	-81.36578
KO6	ON	3	2.15	Y	47.79829	-79.63181
LO3	ON	3	3.41	Y	46.87602	-79.76968
NP3	ON	3	6.06	Y	49.01458	-88.18948
NY1	ON	3	3.41	Y	48.79333	-86.58409
St Joe	ON	3	3.31	Y	46.24400	-83.93700
Trout Lake	ON	4	3.11	Y	46.63427	-84.29080
WR1	ON	3	4.23	Y	48.61890	-85.33645

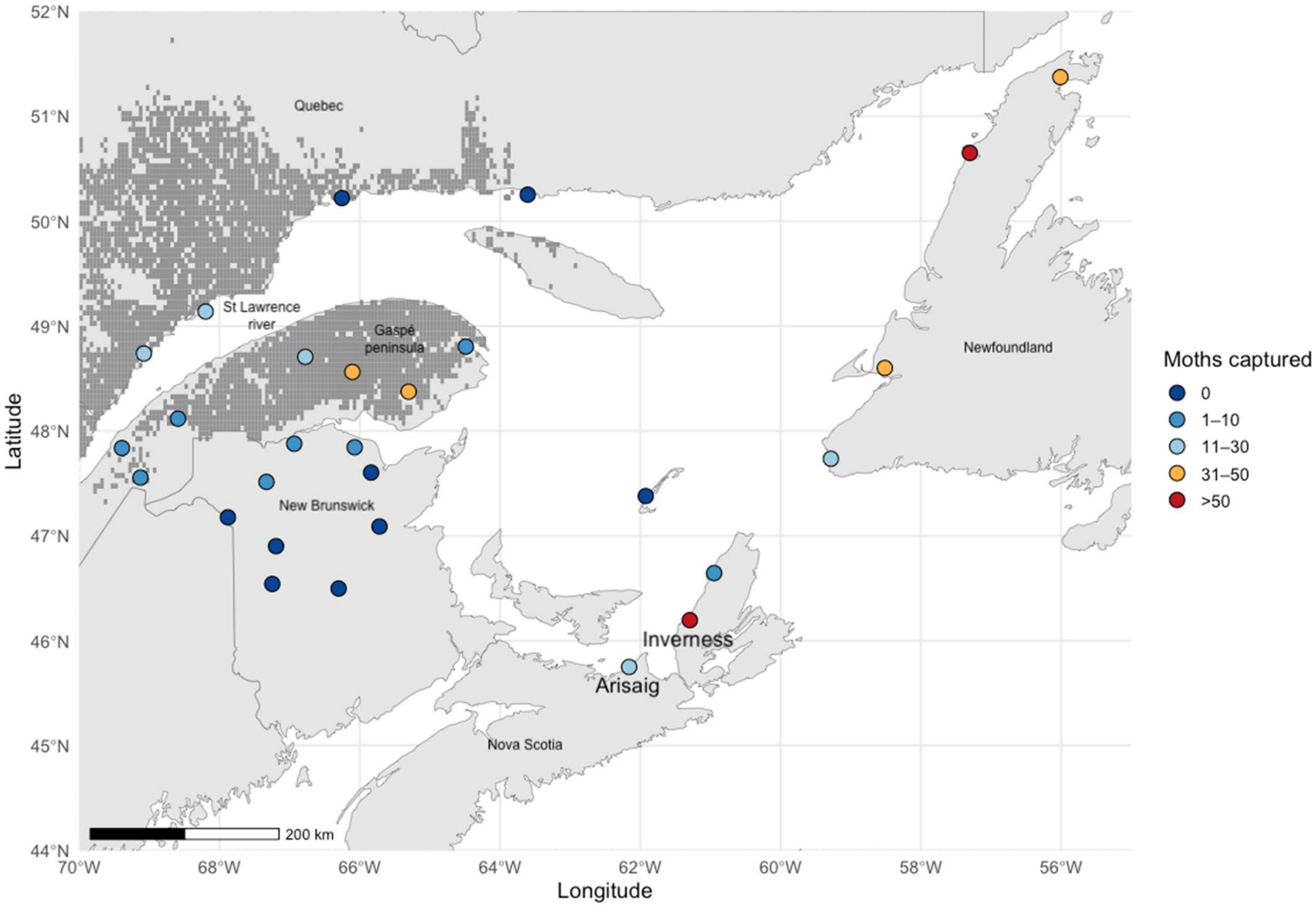

Figure S3: Moth captures in the eastern Canada trap network on the night of July, 22nd-23rd 2020. Points represent trap locations and are colour-coded to represent the range of individual moths captured. Spruce budworm defoliated areas in 2020 (Ministère des Forêts 2020) are coloured as dark grey pixels.

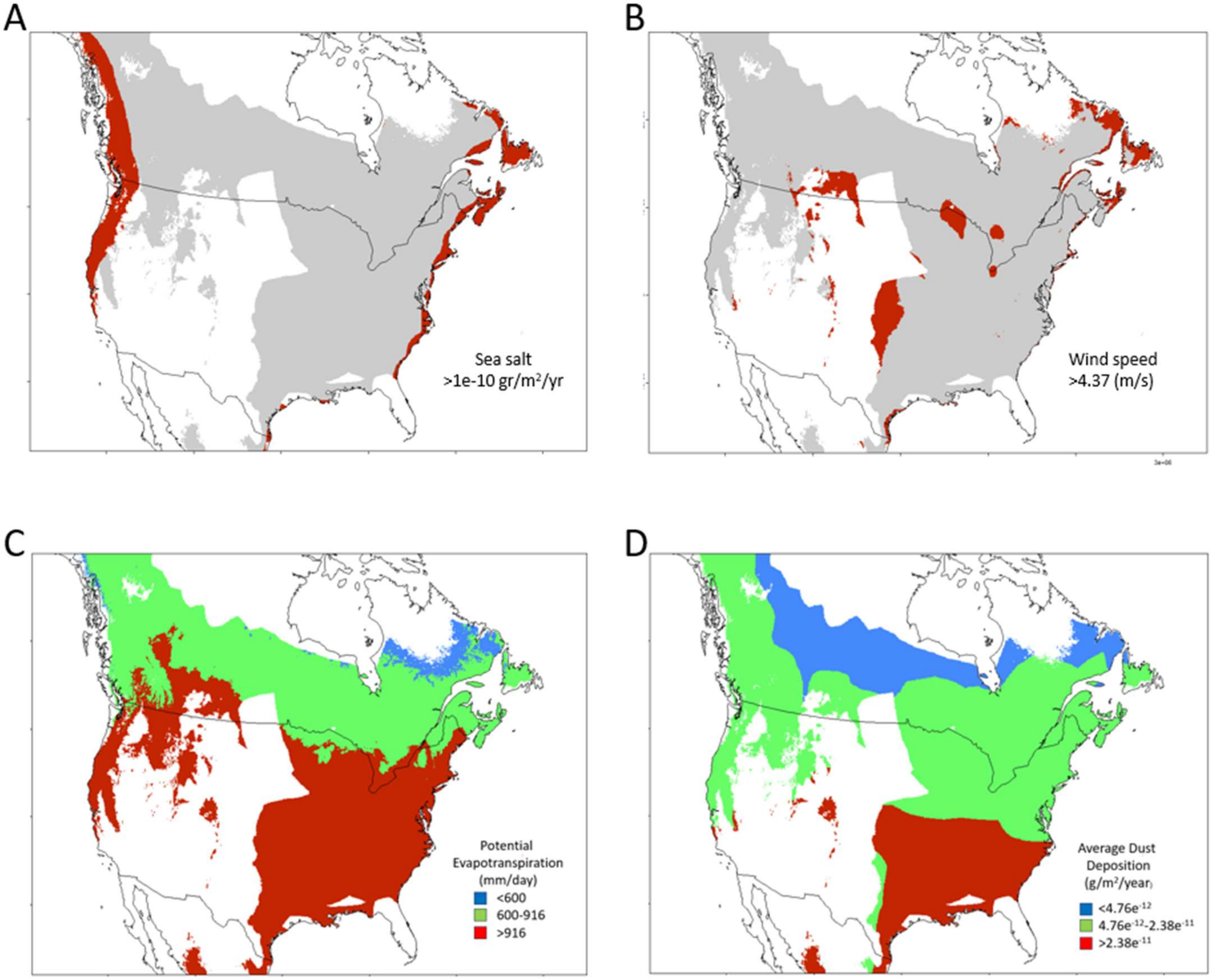

Figure S4: Evaluation of spatial thresholds based on partial dependence plots. (A) sea salt aerosol deposition (g/m^2/yr), (B) wind speed (m/s), (C) potential evapotranspiration (mm/day), and and (D) mineral dust deposition (g/m^2/yr). Values are only provided for areas where all predictors have values within the training set. Border polygons are from *rnaturalearth* (Massicotte & South, 2023)

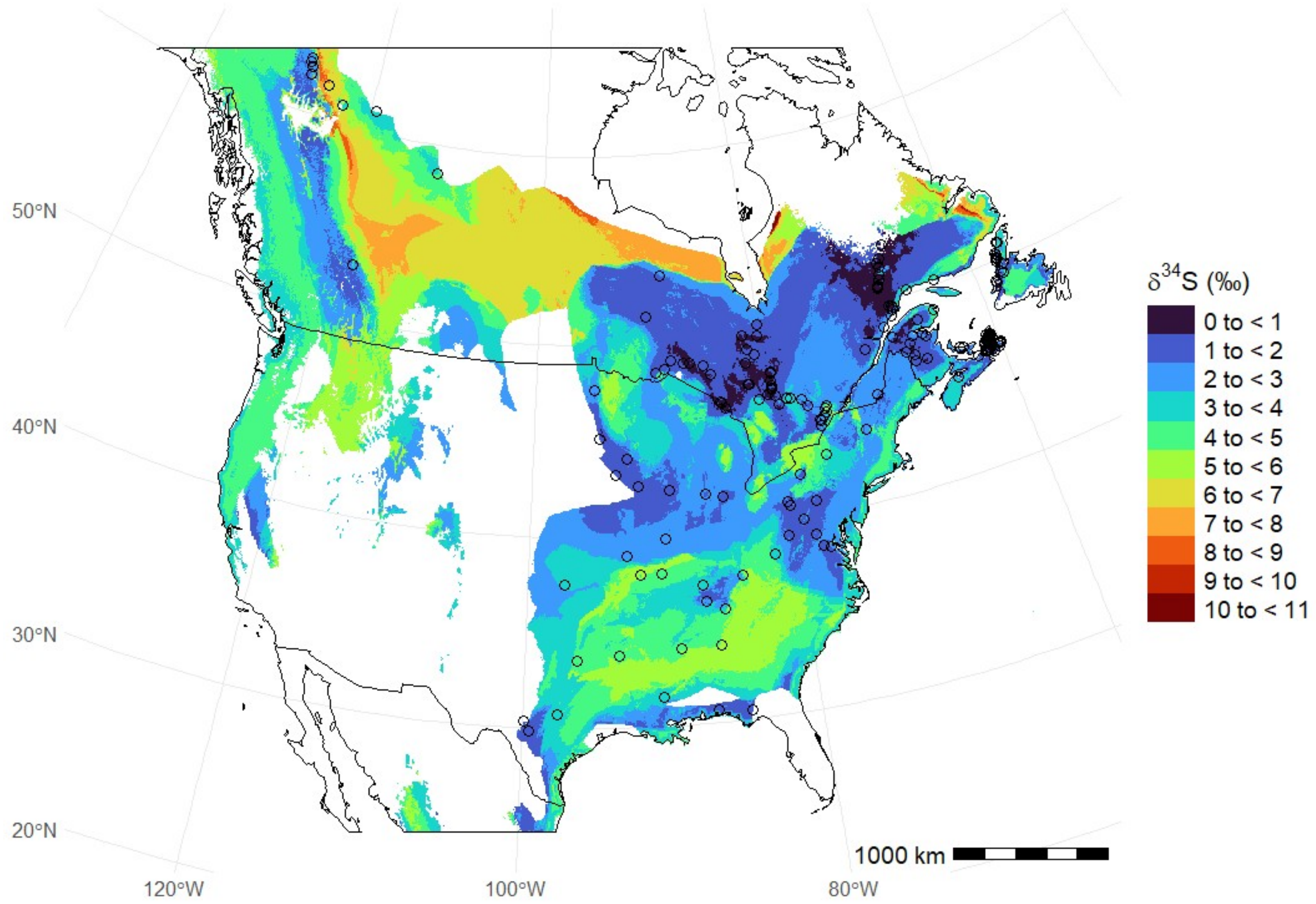

Figure S5: Spatially-explicit uncertainty from the random forest predictions. The uncertainty represents one standard deviation and was obtained from quantile random forest modeling (see Methods). Empty circles indicate locations where foliar samples were collected. As for Figure 2, predictions are only provided for areas where predictors have values within the training set. Border polygons are from *rnaturalearth* (Massicotte & South, 2023).

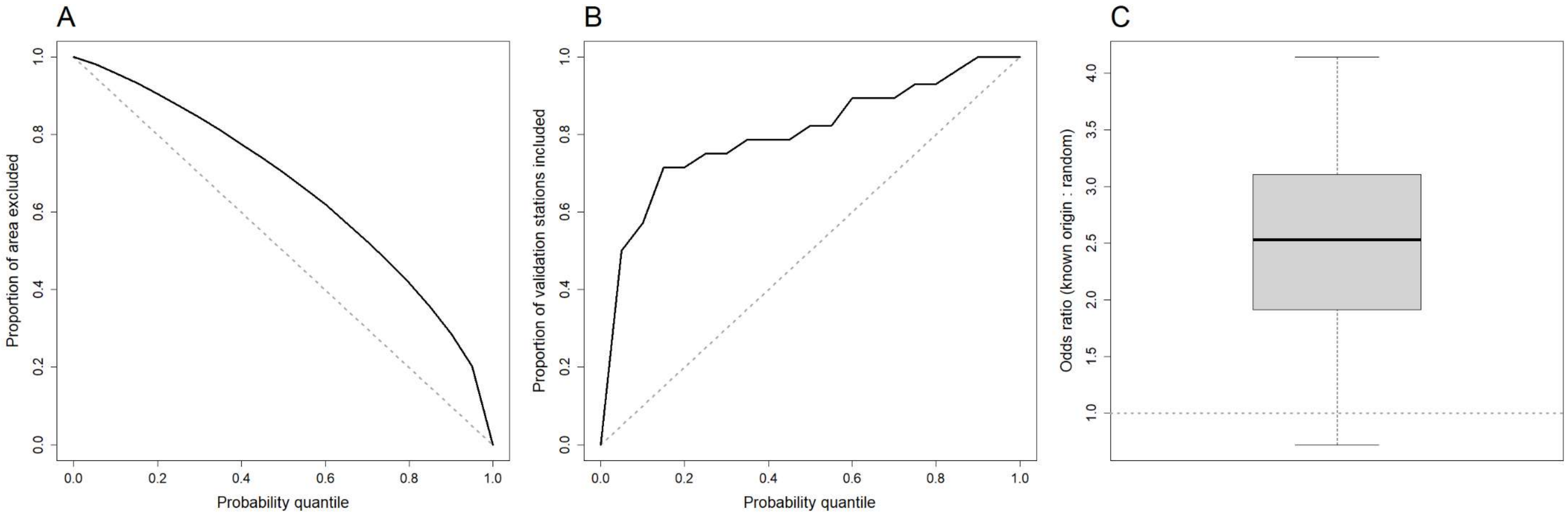

Figure S6: Quality assessment plot of $\delta^{34}S_{moth}$ isoscape using known-origin spruce budworm moth. The *QA* function in *calRaster* (Ma et al. 2020) randomly splits the known-origin data into calibration and validation subsets, recalibrates the foliar isoscape using the calibration set and assigns samples from the validation set, repeating this iteratively. (A) A measure of granularity which shows the proportion of study area (Eastern Canada) that is excluded from assignments of origin based of the probability threshold. A higher curve denotes capacity for more precise assignments. (B) A measure of bias, as the proportion of validation samples that are correctly assigned based on the probability threshold. When accurate, the posterior probabilities fall along the 1:1line, higher values suggest overfitting. (C) The posterior probability of all the known-origin locations relative random locations, where higher values indicate a higher odd of an individual being assigned to its known location than a random one, akin to stronger support for a given site relative to others.

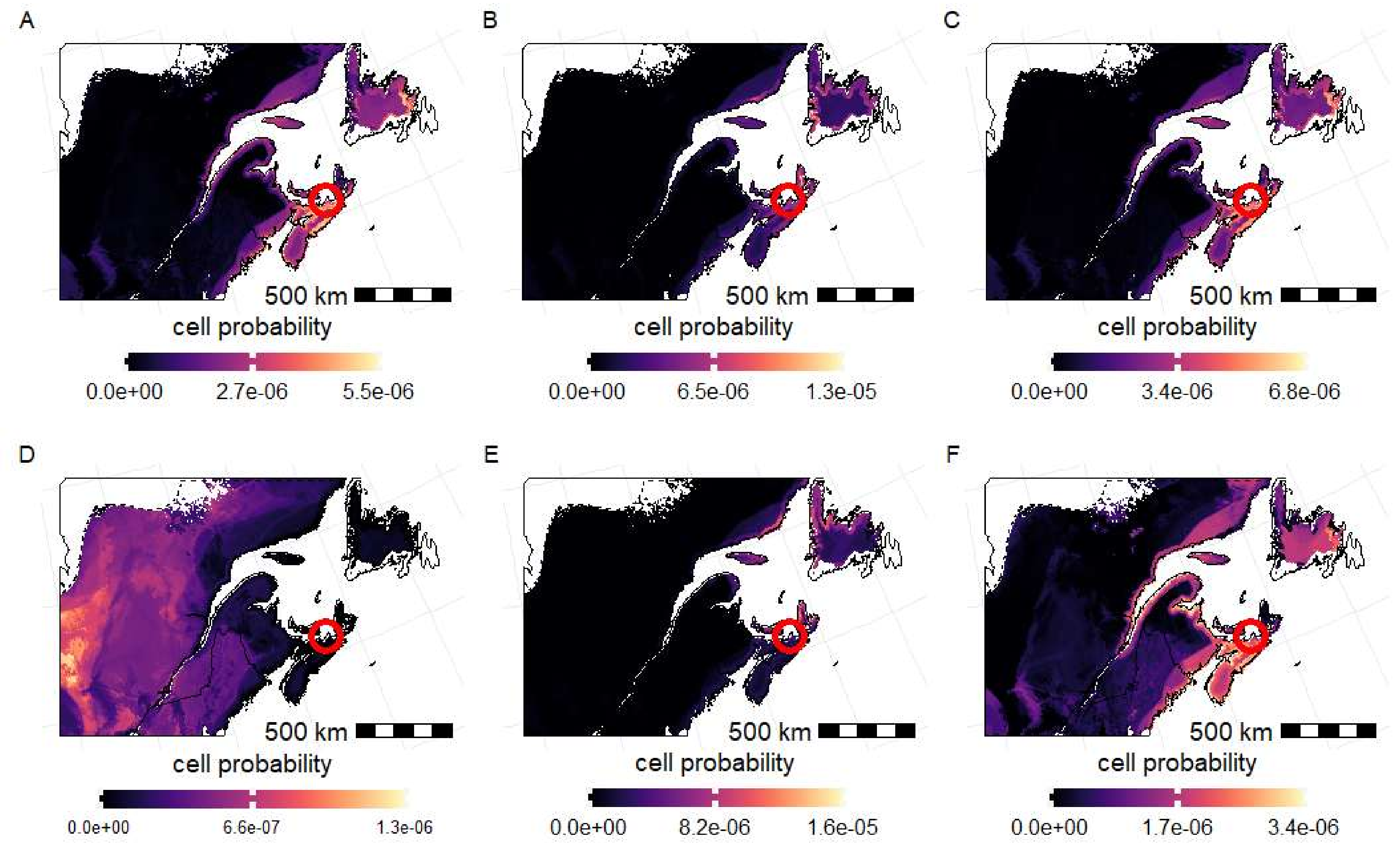

Figure S7: Posterior probability maps of all six individuals sampled from the Arisaig (NS) trap that were classified as local based on capture time inferences (A-F). Bright yellow areas represent sites of higher probability of origin, black areas represent no probability of the tissue having originated there. Trap location shown as red circle. All cells in model sum up to a probability of 1. Values are only provided for areas where all predictors have values within the training set and within the spruce budworm distribution range (broken line). Border polygons are from *rnaturalearth* (Massicotte & South, 2023). Five of the individuals have a δ^{34}S value similar to that of their capture area, confirming the classification of local (A-C, E-F). However, one individual had a δ^{34}S value that suggests that it originated from a non-local area (D).

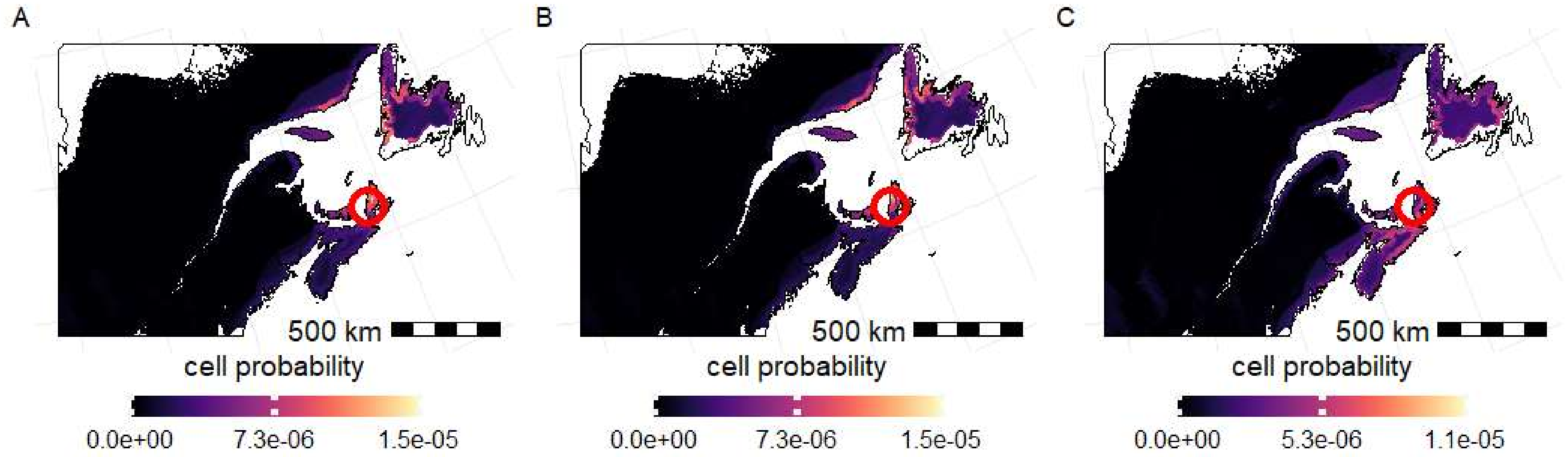

Figure S8: Posterior probability maps of all three individuals from the Inverness (NS) trap that were classified as local based on capture time inferences (A-C). Bright yellow areas represent sites of higher probability of origin, black areas represent no probability of the tissue having originated there. Trap location shown as red circle. All cells in model sum up to a probability of 1. Values are only provided for areas where all predictors have values within the training set and within the spruce budworm distribution range (broken line). Border polygons are from *rnaturalearth* (Massicotte & South, 2023). All individuals have a δ^{34}S value similar to that of their capture area, confirming the classification of local.

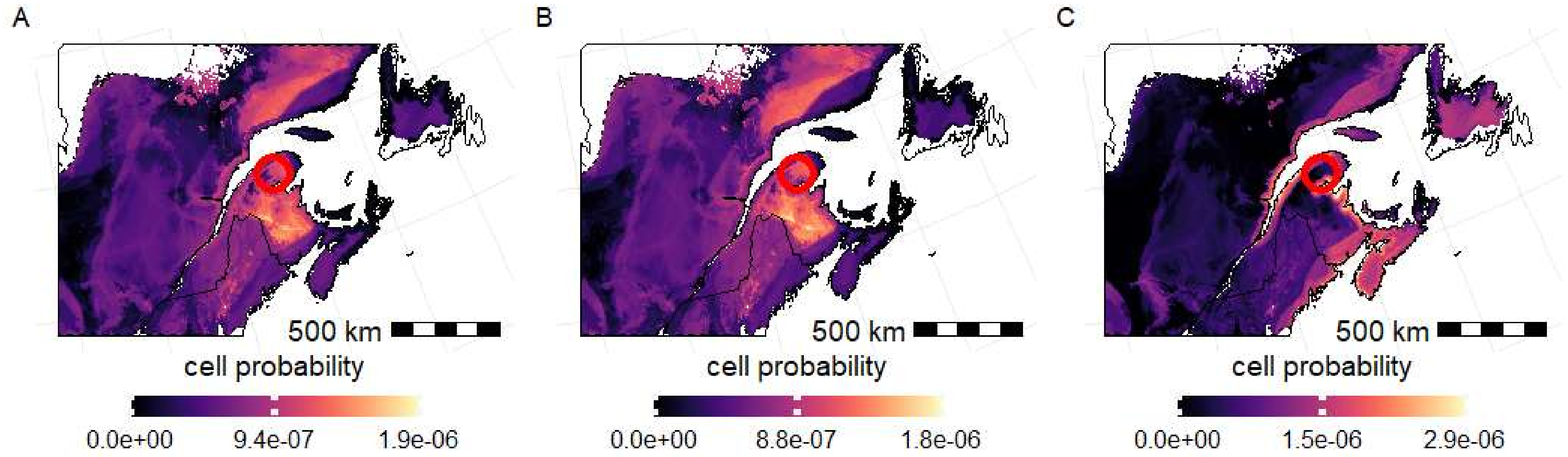

Figure S9: Posterior probability maps of all three individuals from the Baldwin (QC) trap that were classified as local based on capture time inferences (A-C). Bright yellow areas represent sites of higher probability of origin, black areas represent no probability of the tissue having originated there. Trap location shown as red circle. All cells in model sum up to a probability of 1. Values are only provided for areas where all predictors have values within the training set and within the spruce budworm distribution range (broken line). Border polygons are from *rnaturalearth* (Massicotte & South, 2023). All of the individuals have a δ^{34}S value similar to that of their capture area, confirming the classification of local.

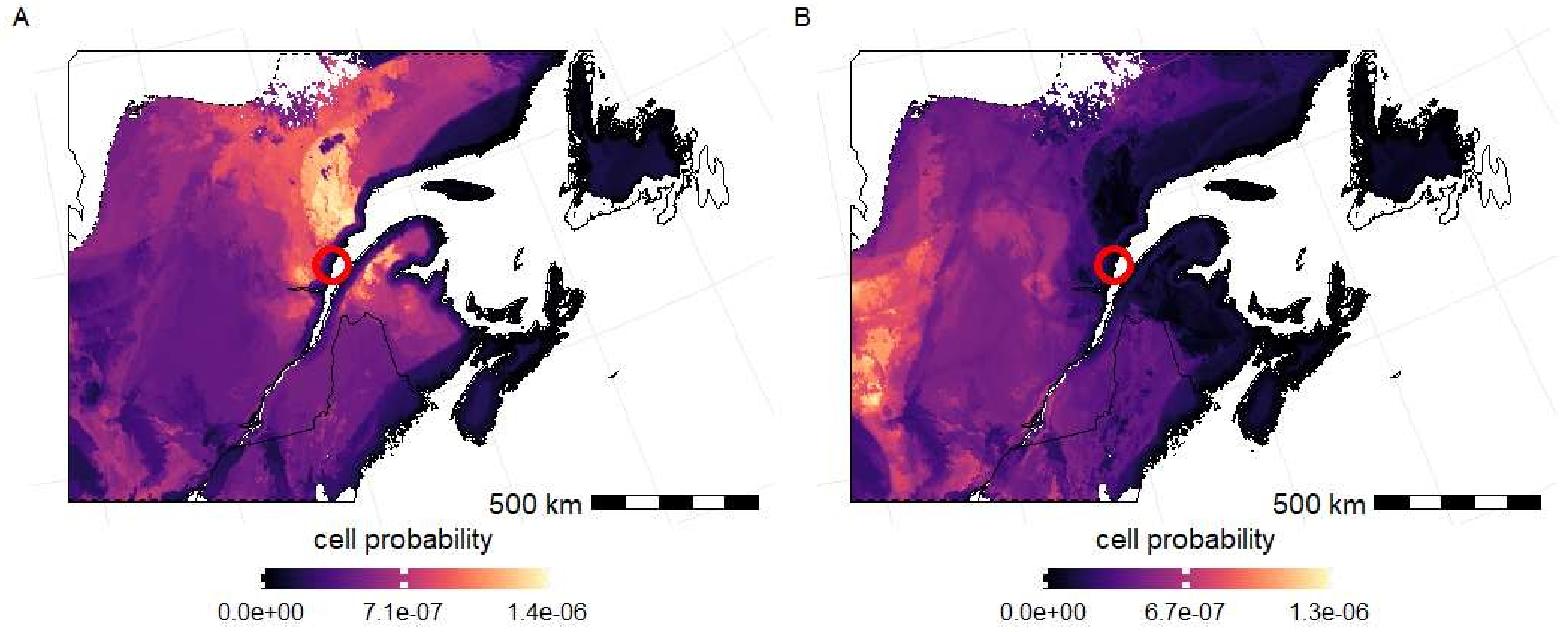

Figure S10: Posterior probability maps of all two individuals the Forestville (QC) trap that were classified as local based on capture time inferences (A-B). Bright yellow areas represent sites of higher probability of origin, black areas represent no probability of the tissue having originated there. Trap location shown as red circle. All cells in model sum up to a probability of 1. Values are only provided for areas where all predictors have values within the training set and within the spruce budworm distribution range (broken line). Border polygons are from *rnaturalearth* (Massicotte & South, 2023). Individuals A and B, both have values that could originate in their capture area, but with low probability.

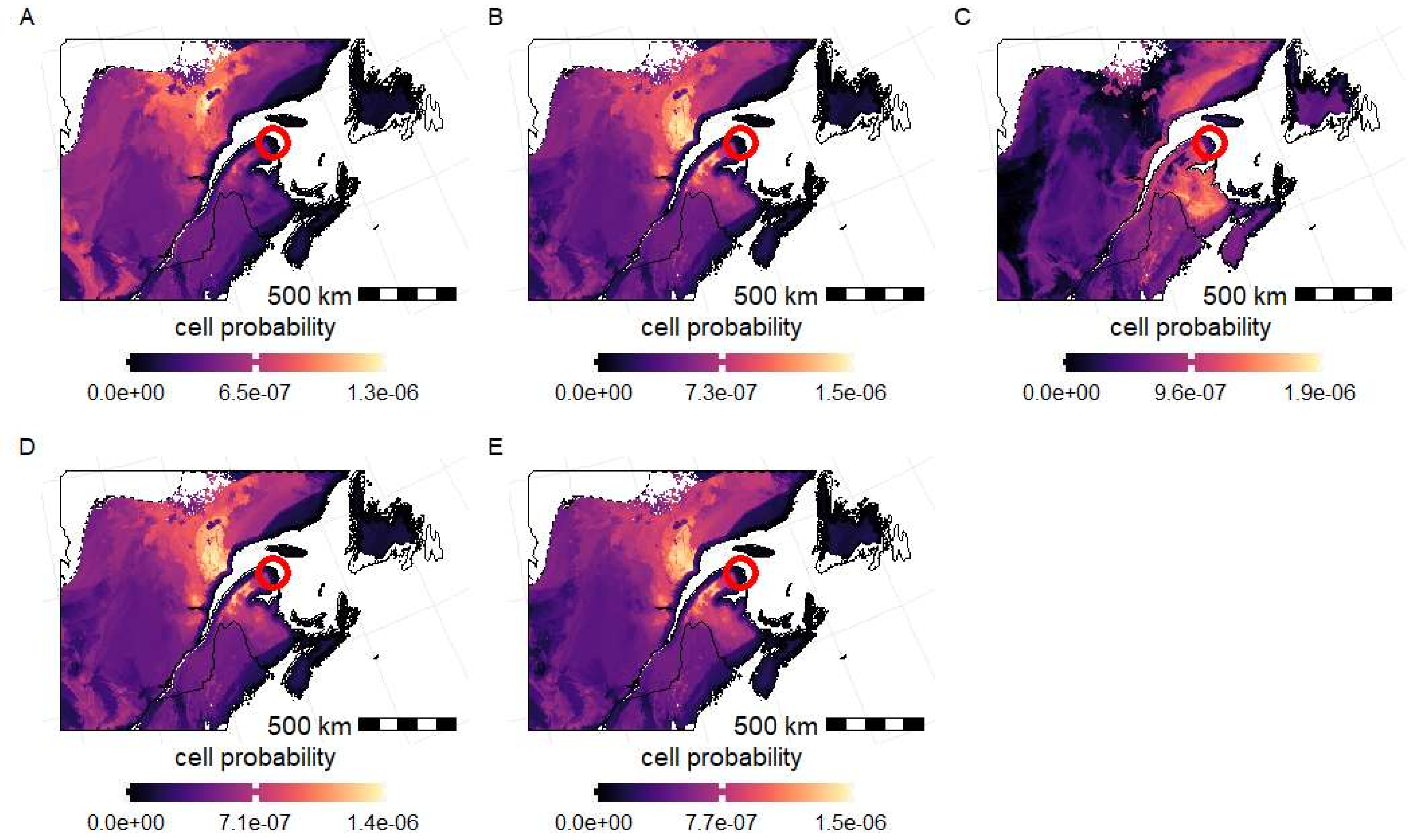

Figure S11: Posterior probability maps of all three individuals from the Gaspe (QC) trap that were classified as local based on capture time inferences (A-C), with a duplicate of individual A using the same tissues (i.e. head and thorax) (D), and another duplicate of A using abdomen tissue (E). Bright yellow represent sites of higher probability of origin, clack areas represent no probability of the tissue having originated there. Trap location shown as red circle. All cells in model sum up to a probability of 1. Values are only provided for areas where all predictors have values within the training set and within the spruce budworm distribution range (broken line). Border polygons are from *rnaturalearth* (Massicotte & South, 2023). The three individuals (A-C), and the replicates of individual A (D-E), have a δ^{34}S value similar to that of their capture area, but with low probability of origin.

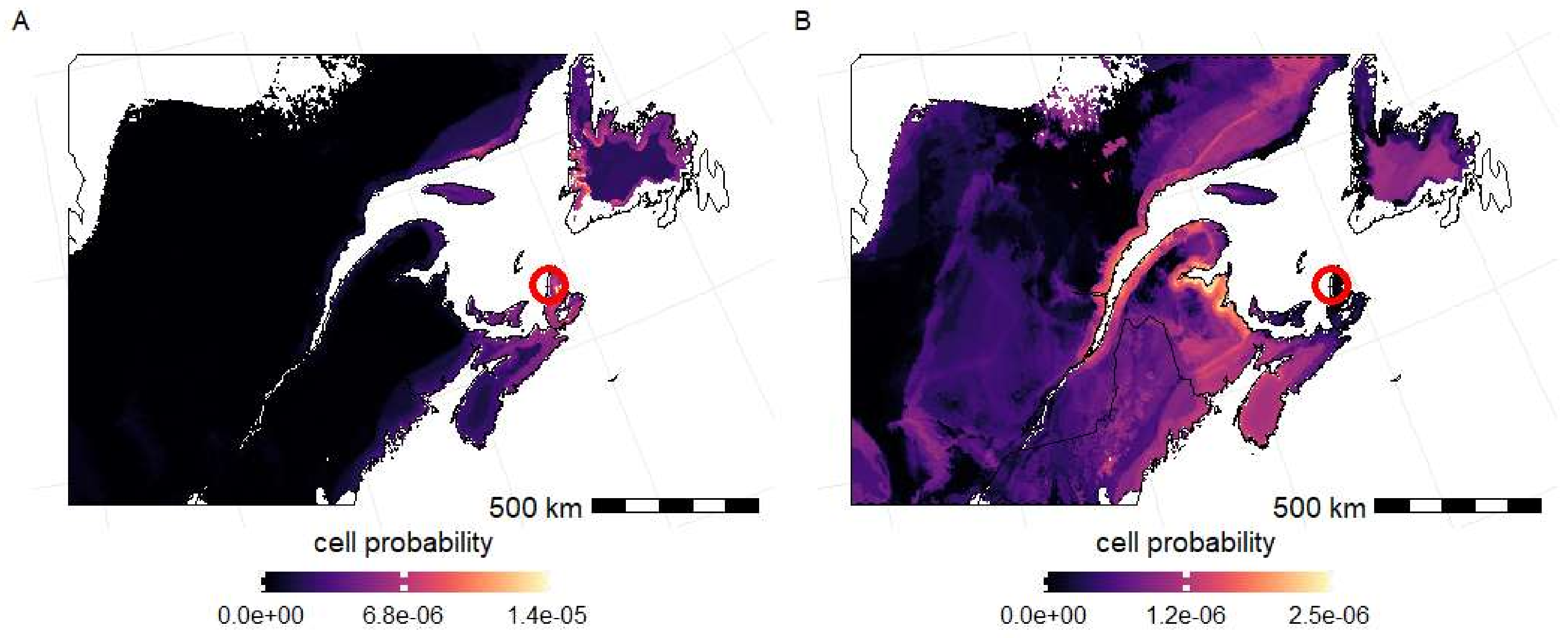

Figure S12: Posterior probability maps of all individuals from the Petit Étang (NS) trap that were classified as local based on capture time inferences (A-B). Bright yellow areas represent sites of higher probability of origin, black areas represent no probability of the tissue having originated there. Trap location shown as red circle. All cells in model sum up to a probability of 1. Values are only provided for areas where all predictors have values within the training set and within the spruce budworm distribution range (broken line). Border polygons are from *rnaturalearth* (Massicotte & South, 2023). Individual A had a $\delta^{34}S$ value similar to that of its capture area, confirming the classification of local, whereas individual B had a $\delta^{34}S$ value that suggests that it originated from a non-local area.

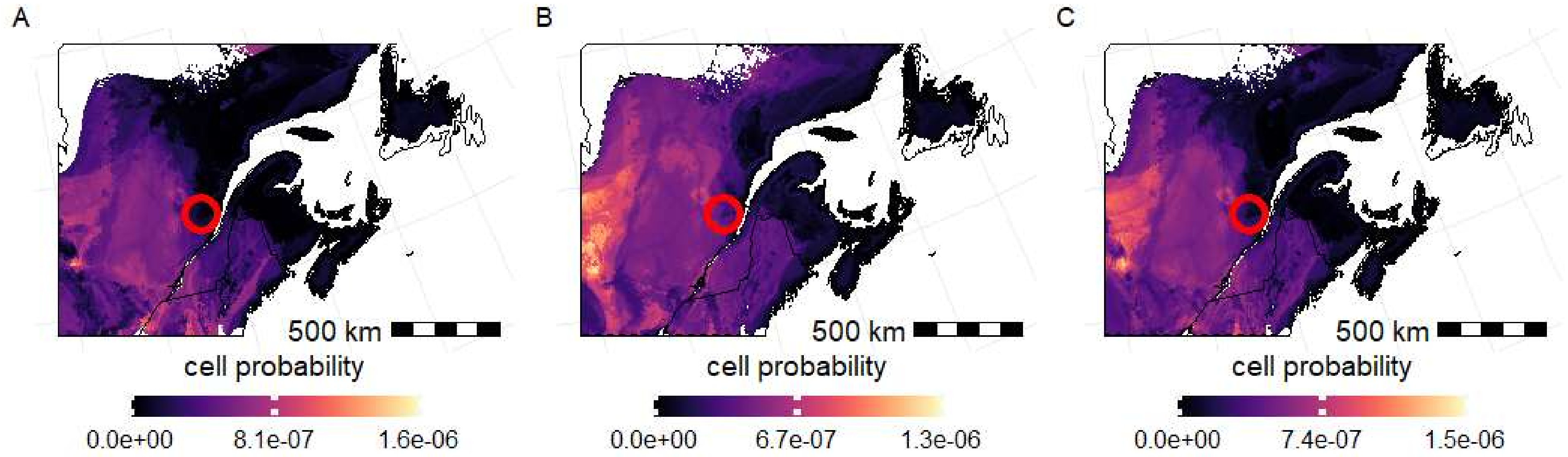

Figure S13: Posterior probability maps of all three individuals from the Pikauba (QC) trap that were classified as local based on capture time inferences (A-C). Bright yellow areas represent sites of higher probability of origin, black areas represent no probability of the tissue having originated there. Trap location shown as red circle. All cells in model sum up to a probability of 1. Values are only provided for areas where all predictors have values within the training set and within the spruce budworm distribution range (broken line). Border polygons are from *rnaturalearth* (Massicotte & South, 2023). Two of the individuals have a δ^{34}S value similar to that of their capture area, confirming the classification of local (B-C). However, one individual had a δ^{34}S value that suggests that it originated from a non-local area (A).

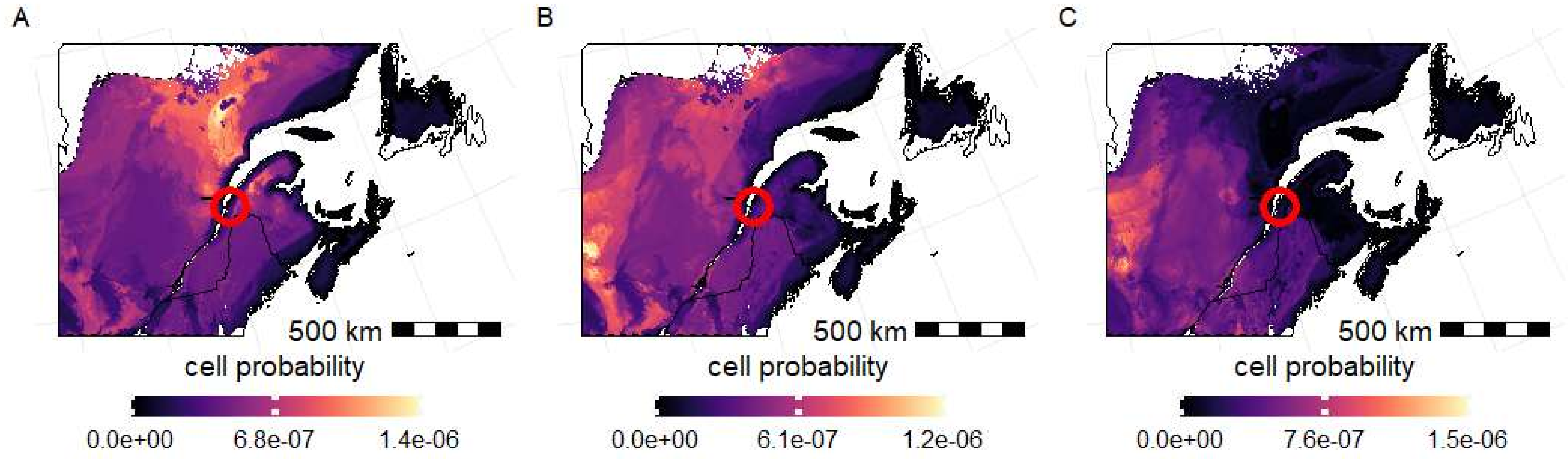

Figure S14: Posterior probability maps of all three individuals from the Sainte Modeste (QC) trap that were classified as local based on capture time inferences (A-C). Bright yellow areas represent sites of higher probability of origin, black areas represent no probability of the tissue having originated there. Trap location shown as red circle. All cells in model sum up to a probability of 1. Values are only provided for areas where all predictors have values within the training set and within the spruce budworm distribution range (broken line). Border polygons are from *rnaturalearth* (Massicotte & South, 2023). All of the individuals have a δ^{34}S value similar to that of their capture area, confirming the classification of local, but one of those individuals has a low probability of origin (C).

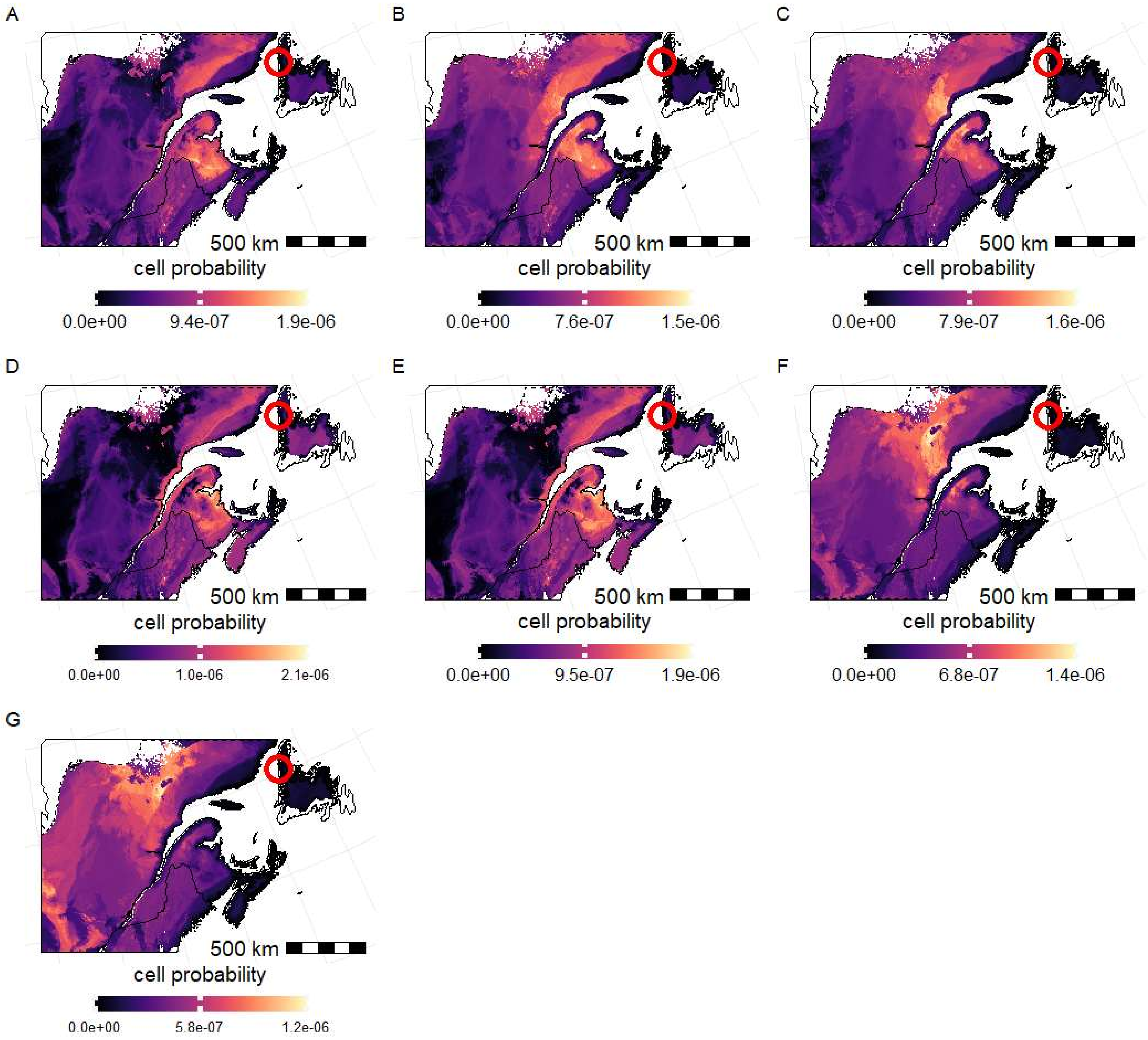
A
500 km
cell probability
0.0e+00
9.4e-07
1.9e-06
B
500 km
cell probability
0.0e+00
7.6e-07
1.5e-06
C
500 km
cell probability
0.0e+00
7.9e-07
1.6e-06
D
500 km
cell probability
0.0e+00
1.0e-06
2.1e-06
E
500 km
cell probability
0.0e+00
9.5e-07
1.9e-06
F
500 km
cell probability
0.0e+00
6.8e-07
1.4e-06
G
500 km
cell probability
0.0e+00
5.8e-07
1.2e-06

Figure S15: Posterior probability maps of all seven individuals from the Zinc Mine road (NL) trap that were classified as local based on capture time inferences (A-G). Bright yellow areas represent sites of higher probability of origin, black areas represent no probability of the tissue having originated there. Trap location shown as red circle. All cells in model sum up to a probability of 1. Values are only provided for areas where all predictors have values within the training set and within the spruce budworm distribution range (broken line). Border polygons are from *rnaturalearth* (Massicotte & South, 2023). Most individuals had a δ^{34}S value that suggests that it originated from a non-local area (B-C, F-G). However, three individuals have a δ^{34}S value similar to that of their capture area, albeit with low probability (A, D-E).

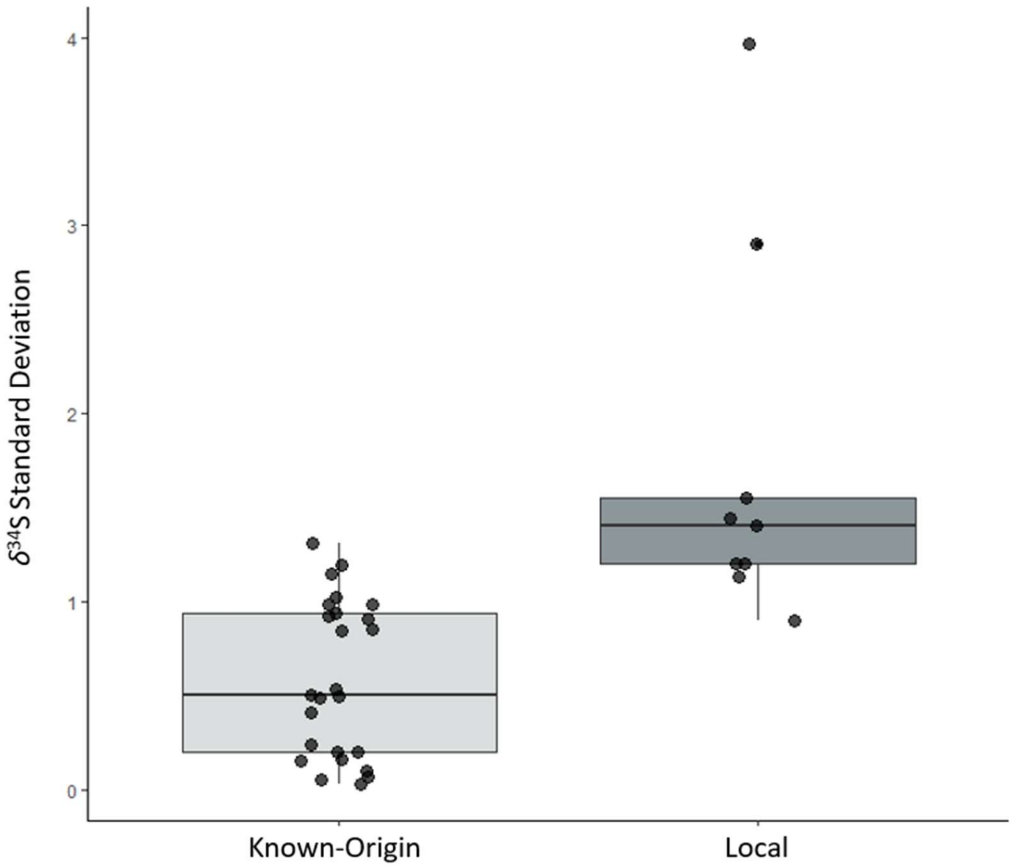

Figure S16: Box and whiskers plot of known origin (i.e., moths collected as pupae) and putative locals (i.e., inferred by time and date of capture on automated traps) within-site $\delta^{34}S_{moth}$ variation. Each point represents the within-site standard deviation in $\delta^{34}S_{moth}$ value among individuals. Mean standard deviation (known-origin: 0.59 ‰, locals: 1.74‰) was significantly different between groups ($t_{8.99}$ = -3.33, p = 0.009).

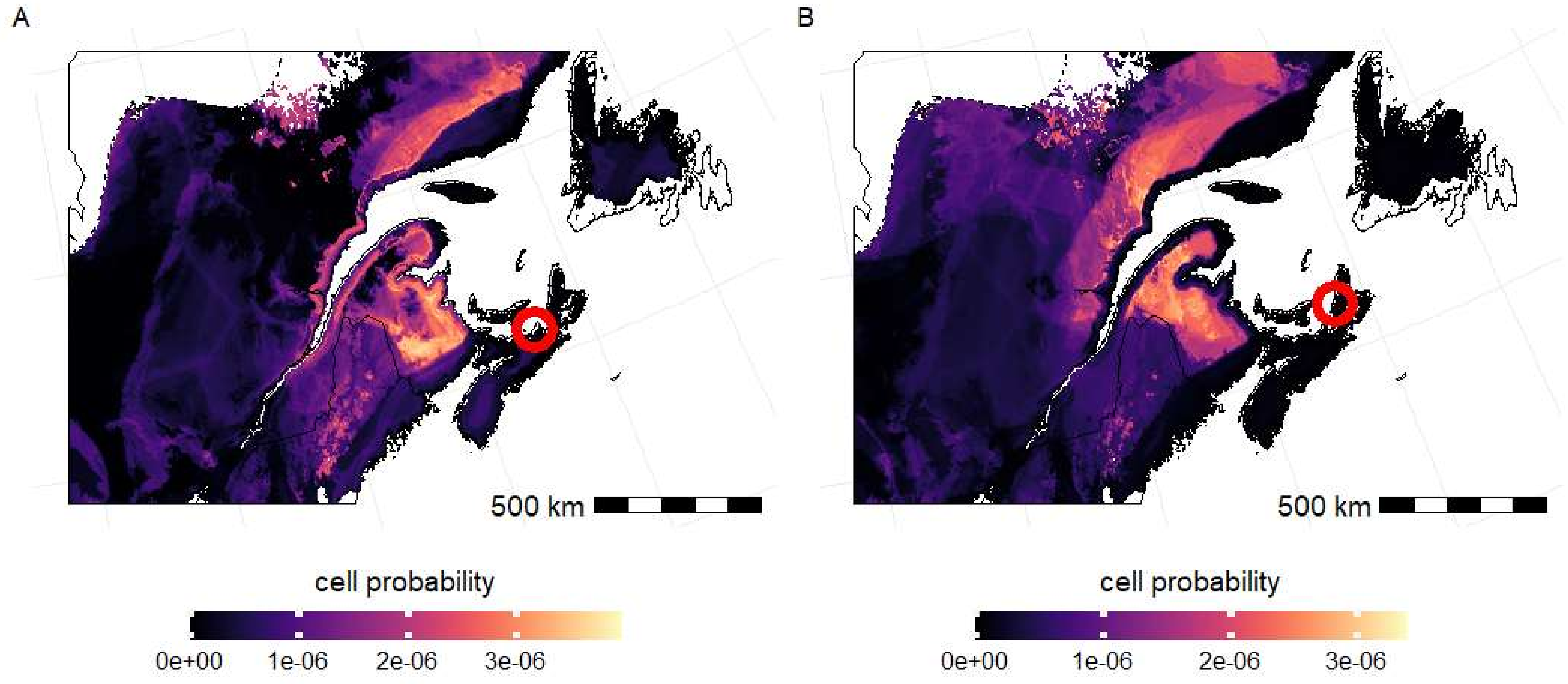

Figure S17: Joint posterior probability of origin map for all immigrants sampled from Arisaig (A, n=3) and Inverness (B, n=3). Joint probability corresponds to the probability of all three individuals coming from each grid cell in the analysis area, bright yellow values indicate higher probability of origin in a given cell, whereas black values indicate no probability of origin. Trap location shown as red circle. All cells in model sum up to a probability of 1. Values are only provided for areas where all predictors have values within the training set and within the spruce budworm distribution range (broken line). Border polygons are from *rnaturalearth* (Massicotte & South, 2023).

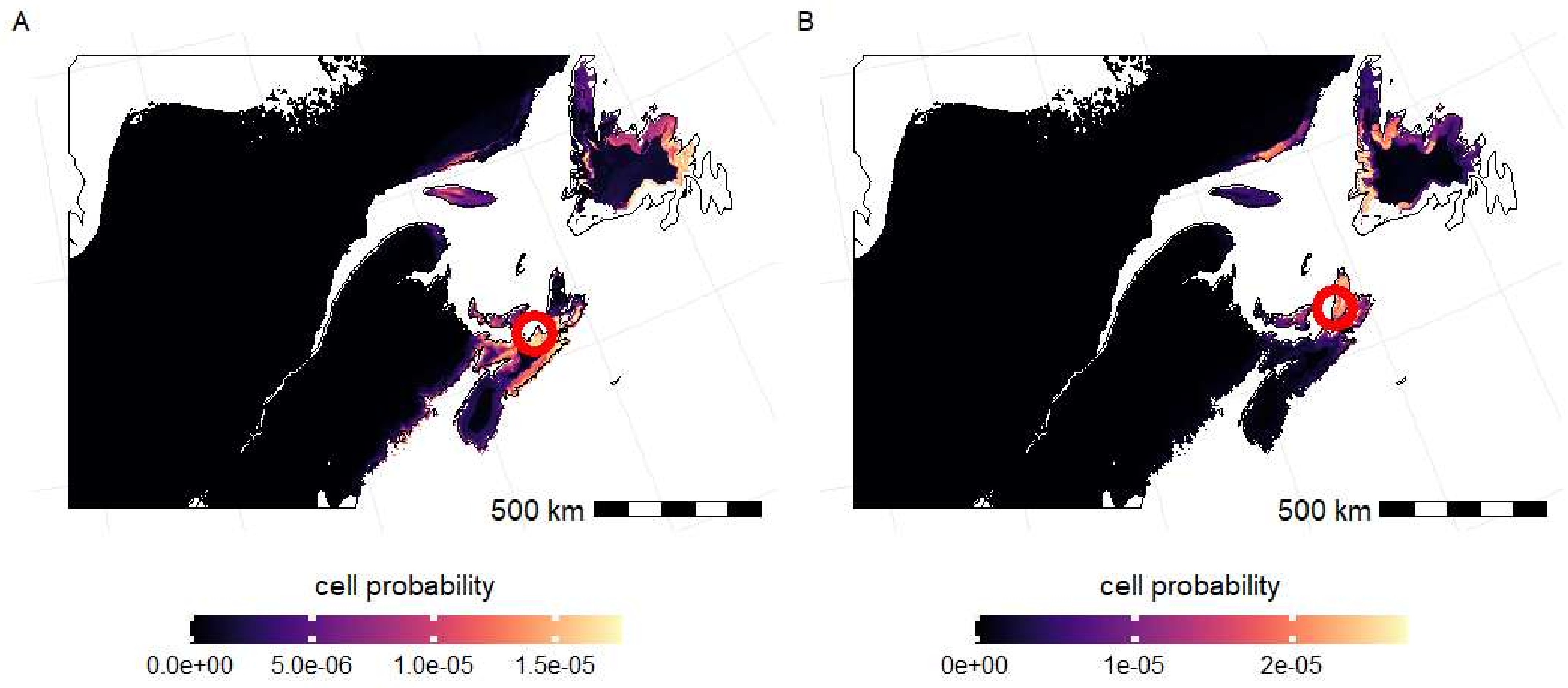

Figure S18: Joint posterior probability of origin map for five out of six locals captured at Arisaig (A) and all three locals capture at Inverness (B). Joint probability corresponds to the probability of all individuals coming from each grid cell in the analysis area, bright yellow values indicate higher probability of origin in a given cell, whereas black values indicate no probability of origin. Trap location shown as red circle. All cells in model sum up to a probability of 1. Values are only provided for areas where all predictors have values within the training set and within the spruce budworm distribution range (broken line). Border polygons are from *rnaturalearth* (Massicotte & South, 2023).

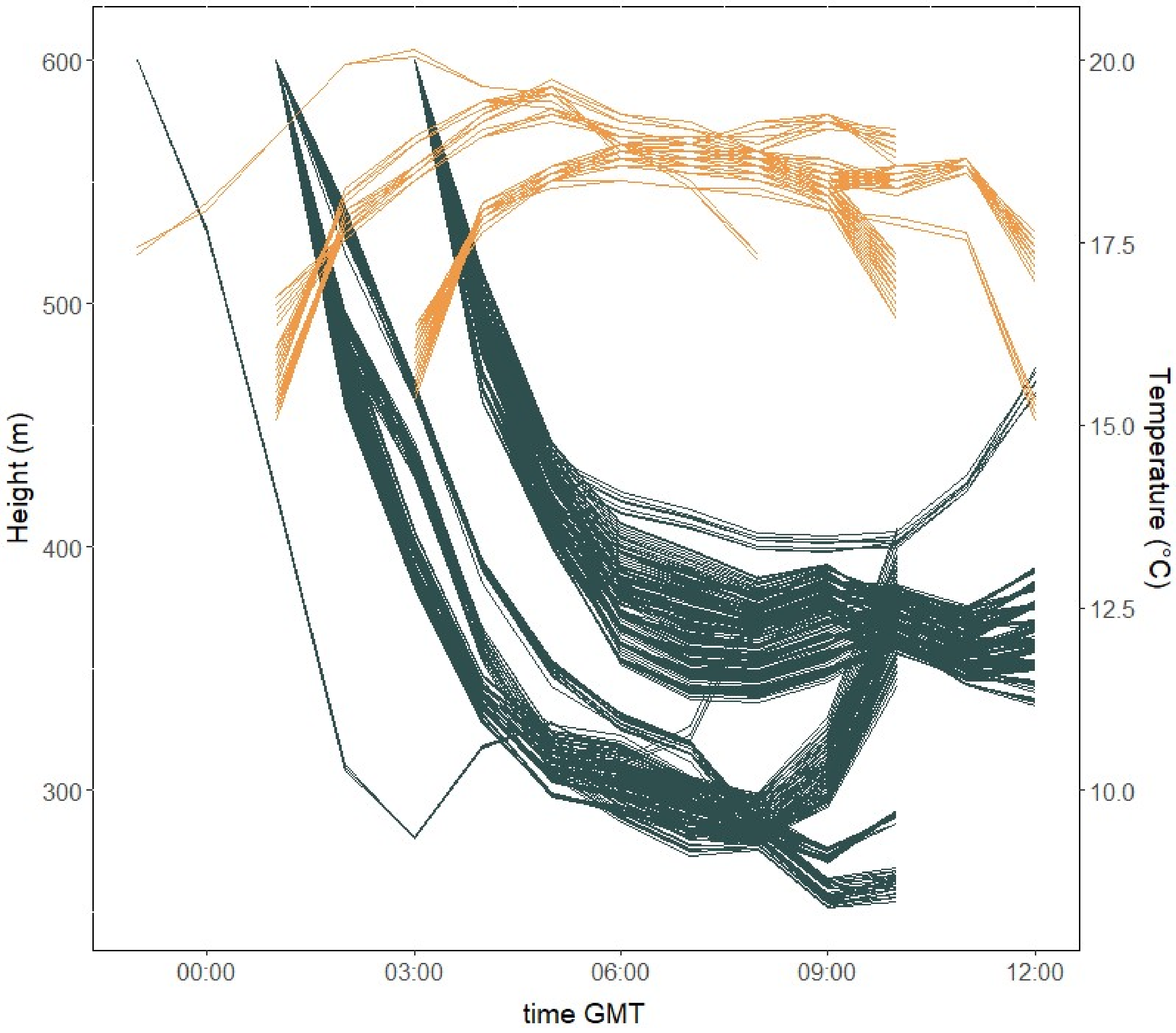

Figure S19: Altitude and temperature along the trajectories simulated using HYSPLIT. Lines represent each individual trajectory for altitude (green, left axis) and temperature (orange, right axis). Time is on GMT, simulations were started at equivalent 19 h, 21 h, and 23 h eastern time, and allowed to continue for nine hours.

References:

Balmino, G., N. Vales, S. Bonvalot, and A. Briais. 2012. Spherical harmonic modelling to ultra-high degree of Bouguer and isostatic anomalies. Journal of Geodesy **86**:499-520.

Bataille, C. P., B. E. Crowley, M. J. Wooller, and G. J. Bowen. 2020. Advances in global bioavailable strontium isoscapes. Palaeogeography, Palaeoclimatology, Palaeoecology **555**:109849.

Bataille, C. P., K. Jaouen, S. Milano, M. Trost, S. Steinbrenner, É. Crubézy, and R. Colleter. 2021. Triple sulfur-oxygen-strontium isotopes probabilistic geographic assignment of archaeological remains using a novel sulfur isoscape of western Europe. Plos One **16**:e0250383.

Bataille, C. P., I. C. C. von Holstein, J. E. Laffoon, M. Willmes, X.-M. Liu, and G. R. Davies. 2018. A bioavailable strontium isoscape for Western Europe: A machine learning approach. Plos One **13**:e0197386.

Börker, J., J. Hartmann, T. Amann, and G. Romero-Mujalli. 2018. Terrestrial sediments of the Earth: Development of a global unconsolidated sediments map database (GUM). Geochemistry, Geophysics, Geosystems **19**:997-1024.

Böttcher, M. E., H. J. Brumsack, and C. D. Dürselen. 2007. The isotopic composition of modern seawater sulfate: I. Coastal waters with special regard to the North Sea. Journal of Marine Systems **67**:73-82.

Brahney, J., A. P. Ballantyne, P. Kociolek, P. R. Leavitt, G. L. Farmer, and J. C. Neff. 2015. Ecological changes in two contrasting lakes associated with human activity and dust transport in western Wyoming. Limnology and Oceanography **60**:678-695.

Brlík, V., P. Procházka, L. Bontempo, F. Camin, F. Jiguet, G. Osváth, C. A. Stricker, M. B. Wunder, and R. L. Powell. 2024. Geographic distribution of feather δ^{34}S in Europe. Ecosphere **15**:e4690.

Brlík, V., P. Procházka, B. Hansson, C. A. Stricker, E. Yohannes, R. L. Powell, and M. B. Wunder. 2023. Animal tracing with sulfur isotopes: Spatial segregation and climate variability in Africa likely contribute to population trends of a migratory songbird. Journal of Animal Ecology **92**:1320-1331.

Chien, C. T., K. R. Mackey, S. Dutkiewicz, N. M. Mahowald, J. M. Prospero, and A. Paytan. 2016. Effects of African dust deposition on phytoplankton in the western tropical Atlantic Ocean off Barbados. Global Biogeochemical Cycles **30**:716-734.

Fick, S. E., and R. J. Hijmans. 2017. WorldClim 2: new 1-km spatial resolution climate surfaces for global land areas. International Journal of Climatology **37**:4302-4315.

Guevara, M., M. Taufer, and R. Vargas. 2020. Gap-free global annual soil moisture: 15 km grids for 1991–2018. Earth System Science Data Discussions **2020**:1-65.

Harris, I., T. J. Osborn, P. Jones, and D. Lister. 2020. Version 4 of the CRU TS monthly high-resolution gridded multivariate climate dataset. Scientific data **7**:109.

Harrison, A., and H. Thode. 1958. Mechanism of the bacterial reduction of sulphate from isotope fractionation studies. Transactions of the Faraday Society **54**:84-92.

Hartmann, J., and N. Moosdorf. 2012. The new global lithological map database GLiM: A representation of rock properties at the Earth surface. Geochemistry, Geophysics, Geosystems **13**.

Jarvis, A., E. Guevara, H. Reuter, and A. Nelson. 2008. Hole-filled SRTM for the globe: version 4: data grid.

Jones, E. L., A. J. Hodson, S. F. Thornton, K. R. Redeker, J. Rogers, P. M. Wynn, T. J. Dixon, S. H. Bottrell, and H. B. O'Neill. 2020. Biogeochemical processes in the active layer and permafrost of a high Arctic fjord valley. Frontiers in Earth Science **8**:1-20.

Lehner, B., and P. Döll. 2004. Development and validation of a global database of lakes, reservoirs and wetlands. Journal of Hydrology **296**:1-22.

Massicotte, P., and A. South. 2023. rnaturalearth: World Map Data from Natural Earth.

Ministère des Forêts, d. l. F. e. d. P. 2020. Aires infestées par la tordeuse des bourgeons de l'épinette au Québec en 2020. Page 31. Gouvernement du Québec, Québec.

Mooney, W. D., G. Laske, and T. G. Masters. 1998. CRUST 5.1: A global crustal model at 5° × 5°. Journal of Geophysical Research: Solid Earth **103**:727-747.

Nehlich, O. 2015. The application of sulphur isotope analyses in archaeological research: A review. Earth-Science Reviews **142**:1-17.

Newton, J. 2021. An insect isoscape of UK and Ireland. Rapid Commun Mass Spectrom **35**:e9126.
Obu, J., S. Westermann, A. Kääb, and A. Bartsch. 2018. Ground temperature map, 2000-2016, Northern hemisphere permafrost. PANGAEA.
Orem, W., C. Gilmour, D. Axelrad, D. Krabbenhoft, D. Scheidt, P. Kalla, P. McCormick, M. Gabriel, and G. Aiken. 2011. Sulfur in the south Florida ecosystem: Distribution, sources, biogeochemistry, impacts, and management for restoration. Critical Reviews in Environmental Science and Technology **41**:249-288.
Poggio, L., L. M. De Sousa, N. H. Batjes, G. B. Heuvelink, B. Kempen, E. Ribeiro, and D. Rossiter. 2021. SoilGrids 2.0: producing soil information for the globe with quantified spatial uncertainty. Soil **7**:217-240.
Sparks, J. M., B. E. Crowley, M. G. Rutherford, and D. Jaggernauth. 2019. Coastal proximity, orientation, and precipitation amount drive spatial variability in delta S-34 values on the Caribbean island of Trinidad. Applied Geochemistry **108**.
Stevens, R. E., H. Reade, K. L. Sayle, J. A. Tripp, D. Frémondeau, A. Lister, I. Barnes, M. Germonpré, M. Street, J. B. Murton, S. H. Bottrell, D. H. James, and T. F. G. Higham. 2025. Major excursions in sulfur isotopes linked to permafrost change in Eurasia during the last 50,000 years. Nature Geoscience **18**:961-965.
Tcherkez, G., and I. Tea. 2013. $^{32}S/^{34}S$ isotope fractionation in plant sulphur metabolism. New Phytologist **200**:44-53.
Trabuco, A., and R. Zomer. 2019. Global aridity index and potential evapotranspiration (ET0) climate database v2. Fileset **10**:m9.
Trust, B. A., and B. Fry. 1992. Stable sulfur isotopes in plants - A review. Plant Cell and Environment **15**:1105-1110.
